# *TP53*‐Mutated Acute Myeloid Leukemia: Unanswered Questions

**DOI:** 10.1002/hon.70106

**Published:** 2025-06-03

**Authors:** Antonella Bruzzese, Ernesto Vigna, Enrica Antonia Martino, Caterina Labanca, Giulio Caridà, Francesco Mendicino, Eugenio Lucia, Virginia Olivito, Noemi Puccio, Antonino Neri, Fortunato Morabito, Massimo Gentile

**Affiliations:** ^1^ Hematology Unit Department of Onco‐Hematology AO of Cosenza Cosenza Italy; ^2^ Department of Experimental and Clinical Medicine University of Catanzaro Catanzaro Italy; ^3^ Laboratory of Translational Reserach Azienda USL‐IRCSS di Reggio Emilia Reggio Emilia Italy; ^4^ Scientific Directorate Azienda USL‐IRCCS di Reggio Emilia Reggio Emilia Italy; ^5^ Gruppo Amici Dell'Ematologia Foundation‐GrADE Reggio Emilia Italy; ^6^ Department of Pharmacy Health and Nutritional Science University of Calabria Rende Italy

**Keywords:** AML, therapy, TP53

## Abstract

*TP53*‐mutated acute myeloid leukemia (AML) remains one of the most treatment‐resistant hematologic malignancies, with poor overall survival despite advancements in therapeutic strategies. The loss of functional p53 compromises DNA repair, apoptosis, and genomic stability, rendering both conventional and novel therapies largely ineffective. This review evaluates the efficacy of various treatment approaches, including intensive chemotherapy (IC), hypomethylating agents (HMAs), venetoclax‐based regimens, and immune checkpoint inhibitors. Additionally, we discuss emerging strategies such as p53 reactivation, multi‐targeted inhibition, and novel immunotherapies, including bispecific T‐cell engagers (BiTEs) and CAR‐T cell therapy. Current treatment options provide limited benefits in *TP53*‐mutated AML, with complete remission rates ranging from 13% to 46% and median overall survival of only 6.1–6.5 months. Allogeneic stem cell transplantation (allo‐SCT) offers minimal survival advantage due to high relapse rates. Despite promising preclinical data, checkpoint inhibitors and TIM‐3 blockade have failed to demonstrate significant clinical efficacy, likely due to the immunosuppressive tumor microenvironment. Novel approaches, such as APR‐246 (eprenetapopt) and MCL‐1/CHK1 inhibitors, are under investigation, but their therapeutic impact remains uncertain. The failure of single‐agent therapies underscores the need for combination strategies targeting multiple resistance mechanisms. Future research should focus on integrating targeted inhibitors with immunotherapy and bone marrow microenvironment modifiers. While *TP53*‐mutated AML remains a formidable challenge, ongoing advances in precision medicine and immunotherapy hold the potential to improve patient outcomes.

## 1 Introduction

Acute myeloid leukemia (AML) encompasses a heterogeneous group of aggressive hematological malignancies characterized by the uncontrolled expansion of myeloid blasts [1, 2, 3, 4, 5].

The therapeutic approach for AML is primarily determined by patient fitness and the biological characteristics of the disease [3, 4, 6].

Recent classifications of AML, including those outlined by the International Consensus Classification (ICC) and the World Health Organization (WHO), have placed increasing emphasis on the molecular landscape of the disease, refining AML subtypes and incorporating novel genetic entities into the classification of myeloid malignancies [3, 4].

To date, more than 5000 driver mutations have been identified in AML, involving genes associated with RNA‐splicing, chromatin remodeling, cellular differentiation, cell cycle regulation, and growth factor signaling [7, 8]. The prognostic significance of individual mutations is frequently influenced by the presence of co‐occurring genetic alterations, underscoring the complexity of AML pathogenesis. Among the various molecular and cytogenetic abnormalities, the presence of chromosomal aneuploidy and *TP53* mutations has been consistently associated with an extremely poor prognosis [9].

However, as we delve deeper into the molecular intricacies of *TP53* mutations, we open a Pandora's box of complexities that complicate treatment and outcomes. Questions arise regarding the best treatment strategies for this highly resistant subpopulation of AML patients. What are the optimal therapeutic regimens for *TP53*‐mutated AML? How do various types of *TP53* mutations influence the efficacy of treatments? Can novel agents, such as immune modulators, targeted inhibitors, and combination therapies, offer hope where traditional therapies fail? This review aims to explore the therapeutic challenges posed by *TP53* mutations in AML, shedding light on the unanswered questions and the promising avenues under investigation. From novel agents in clinical trials to the potential of combination therapies, the landscape of *TP53*‐mutated AML is evolving rapidly. Ultimately, this work will examine whether the treatment of *TP53*‐mutated AML can truly be transformed, or if, as in Pandora's box, the hope for a cure will remain elusive, constrained by the inherent complexities of this aggressive disease.

## 2 Structure and Function of *TP53* in Normal and Pathological Conditions

The *TP53* gene, located on chromosome 17p13.1, is the most frequently mutated in human cancers and plays a fundamental role as a tumor suppressor. Often referred to as the “*guardian of the genome,”* it encodes the p53 transcription factor, which is essential for regulating cell cycle progression, apoptosis, DNA repair, and metabolic homeostasis [7, 8]. While *TP53* mutations are widespread in solid neoplasms, they are detected in approximately 10%–15% of AML cases, with a particularly high prevalence in therapy‐related AML (t‐AML), AML with complex karyotype, and erythroid leukemia [10, 11, 12].

### 2.1 Structural Domains of p53 and DNA‐Binding Mechanisms

Structurally, p53 consists of multiple functional domains that are critical for its activity. At its N‐terminus, p53 contains an intrinsically unfolded transactivation domain (TAD), which is further subdivided into the subdomains TAD1 and TAD2 that mediate interactions with coactivators and regulators. This is followed by a proline‐rich region (PRR) that modulates protein interactions. The central DNA‐binding domain (DBD) is essential for sequence‐specific binding to target genes, while the tetramerization domain (TD) facilitates its assembly into a functional homotetramer. The C‐terminal domain (CTD) regulates p53 stability and activity through various post‐translational modifications [13]. Notably, most *TP53* mutations occur within the DNA‐binding domain, disrupting its ability to recognize and regulate target genes.

**FIGURE 1:**
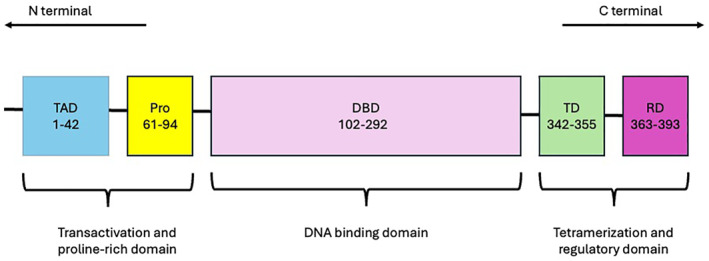
The structure of normal p53 protein.

This domain features a central *β*‐sandwich with an immunoglobulin‐like fold. Its DNA‐binding surface consists of a loop‐sheet‐helix motif and two large loops (L2 and L3), stabilized by a zinc ion [14]. Structural disruptions impair p53's tumor suppressor function, promoting genomic instability and malignant transformation. The structure of normal p53 protein is illustrated in Figure 1.

### 2.2 Regulation of p53 Under Physiological and Stress Conditions

Under normal conditions, p53 is maintained at low levels in both the nucleus and cytoplasm, where its activity is carefully regulated, mainly by the activity of MDM2 that facilitates p53 ubiquitination and subsequent degradation via the 26S proteasomes [15].

However, under conditions of cellular stress—such as DNA damage, hypoxia, or nutrient deprivation—post‐translational modifications, including inhibition of ubiquitination, phosphorylation, acetylation, and methylation, prevent its degradation, leading to p53 accumulation in the nucleus. In this activated state, p53 forms tetrameric complexes that bind DNA and regulate the transcription of more than 150 target genes [16, 17, 18, 19].

Moreover, upon genotoxic stress, p53 activation is triggered by the phosphorylation of key kinases, including ataxia telangiectasia mutated (ATM), ataxia telangiectasia and Rad3‐related (ATR), and casein kinase 2 (CK2) while oncogenic signals driven by MYC or RAS overexpression induce the upregulation of p14ARF, a tumor suppressor that binds MDM2, preventing its inhibitory interaction with p53 [20, 21, 22, 23].

### 2.3 p53‐Mediated Cell Cycle Arrest and DNA Repair Mechanisms

Given its ability to regulate more than 150 target genes, p53 exerts complex and multifaceted cellular functions, many of which remain incompletely understood. However, its role in cell cycle regulation and apoptosis has been extensively characterized [16, 17, 18, 24, 25].

In addition to promoting DNA repair, p53 governs cell cycle progression at two different checkpoints, G1/S and G2/M. The first one is controlled by inducing the transcription of CDKN1A, which encodes p21, a potent cyclin‐dependent kinase (CDK) inhibitor. p21 halts cell cycle progression by preventing the phosphorylation of retinoblastoma protein (Rb) by cyclin D1‐CDK4, cyclin D2‐CDK4, and cyclin E‐CDK2 complexes. In its hypophosphorylated state, Rb sequesters the E2F transcription factor, repressing the expression of genes required for S‐phase entry and enforcing a G1 phase arrest. Beyond G1 regulation, p53 also inhibits the G2/M transition, a critical checkpoint ensuring genomic integrity before mitosis, suppressing the transcription of CDKs and cyclin B, both essential for G2/M phase progression. Furthermore, p53 induces the expression of growth arrest and DNA damage‐inducible 45 protein (Gadd45), which disrupts the cyclin B1/Cdc2 complex, reinforcing the G2 arrest and preventing the propagation of cells with genomic aberrations [25, 26, 27, 28, 29, 30].

### 2.4 p53 and Apoptosis: Intrinsic and Extrinsic Pathways

Beyond cell cycle regulation, p53 functions as a master regulator of apoptosis, orchestrating multiple pathways to eliminate damaged or potentially malignant cells. This pro‐apoptotic role is mediated through many mechanisms: (i) transcriptional activation of key pro‐apoptotic genes which promote mitochondrial outer membrane permeabilization; (ii) direct interaction with anti‐apoptotic proteins antagonizing their inhibitory effects on apoptosis; (iii) activation of Bak, leading to the initiation of the intrinsic apoptotic pathway; (iv) upregulation of miR34a, with a consequent downregulating Bcl‐2; (v) induction of death receptor‐mediated apoptosis enhancing the expression of apoptotic receptors on the cell membrane; and (vi) suppression of transposable elements thereby preserving genomic stability [31, 32, 33, 34, 35, 36, 37, 38, 39, 40, 41].

### 2.5 p53 and Metabolic Regulation in Tumor Suppression

In addition to its role in apoptosis, p53 exerts significant control over cellular metabolism, through different mechanisms: (i) enhancing the transcription of genes involved in oxidative phosphorylation, to promote mitochondrial respiration; (ii) inhibiting glycolysis; (iii) binding to glucose‐6‐phosphate dehydrogenase (G6PDH), thereby suppressing the pentose phosphate pathway and limiting anabolic metabolism; (iv) reducing glucose uptake [42, 43, 44, 45, 46], (v) modulating lipid metabolism [47, 48]; and (vi) regulating nitrogen metabolism [49].

### 2.6 p53 in Ferroptosis and Autophagy Regulation

Furthermore, p53 has emerged as a key modulator of ferroptosis a form of regulated, iron‐dependent cell death characterized by lipid peroxidation thought different mechanisms resulting in a reduced cellular antioxidant capacity, increased glutathione (GSH) biosynthesis, enhanced lipid peroxidation and ferroptosis, reducing its plasma‐membrane‐associated function in lipid peroxidation [50, 51, 52, 53, 54, 55, 56, 57].

Additionally, p53 promotes autophagy through several mechanisms, including upregulation of autophagy‐related genes, enhancing catabolic pathways, and suppressing anabolic growth signals [58, 59, 60, 61, 62, 63, 64, 65, 66].

**FIGURE 2:**
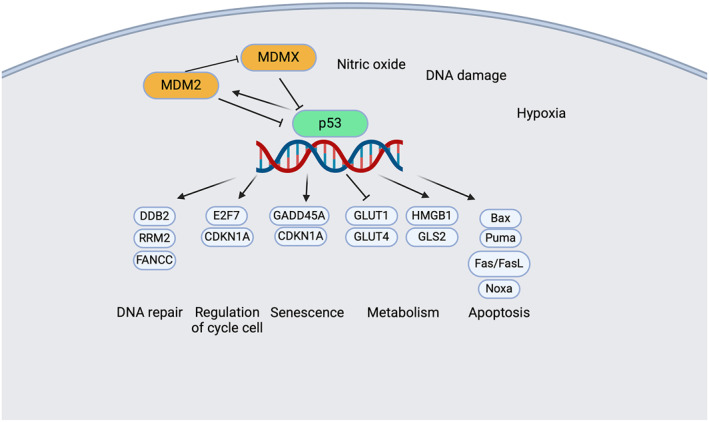
The pleiotropic tumor‐suppressive functions of p53.

The pleiotropic tumor‐suppressive functions of p53, spanning cell cycle regulation, apoptosis, metabolic control, ferroptosis, and autophagy, underscore its pivotal role in maintaining genomic integrity and preventing malignant transformation, as illustrated in Figure 2.

## 3 Role of *TP53* Mutations in AML Pathogenesis

p53 plays a fundamental role in cell cycle regulation and maintenance of cellular homeostasis. Its function is essential for normal hemopoiesis, as disruptions in *TP53* can lead to outgrowth of pluripotent stem cells and subsequent malignant transformation of hematopoietic stem cells [67].

Somatic mutations in *TP53* are associated with aging and are estimated to be present in approximately 4% of preleukemic conditions, such as clonal hemopoiesis of indetermined potential (CHIP). These mutations frequently co‐occur with alterations in other genes, including *DNMT3A, TET2, ASXL1, SRSF2, CBL,* and *SF3B1*. Strong evidence suggests that *TP53* mutations act as an early initiating event, promoting the acquisition of additional genetic abnormalities that facilitate the progression to overt leukemia. However, TP53 mutation alone is generally insufficient for leukemia initiation, necessitating additional oncogenic events [8, 68].

In clinical settings, all *TP53* mutations are often considered collectively, without therapeutic stratification based on specific genotypes. However, a plethora of research has revealed distinct functional subgroups within *TP53* mutations. As previously described, the structure of the p53 protein consists of three main functional domains: the amino‐terminal region (AT), the DNA‐binding domain (DBD), and the oligomerization domain (OD). In AML, most *TP53* mutations occur within the centrally located DNA‐binding domain, followed by the amino‐terminal and oligomerization domains [13, 14].

Mutations in the AT domain are usually insertions or deletions, leading to the full‐length p53 expression. Notably, this region also contains the binding site for p53 negative regulator MDM2 [69]. When full‐length p53 expression is disrupted due to AT domain mutations, an alternative isoform, p47, is produced. This isoform lacks the ability to induce cell‐cycle arrest through CDKN1A, and patients harboring such mutations generally exhibit a comparatively favorable prognosis [70, 71, 72].

The DNA‐binding domain is the most frequently mutated region, accounting for approximately 80% of *TP53* mutations. Alterations in this domain impair the transactivation of multiple target genes, leading to functional inactivation of p53 and a loss‐of‐function (LOF) phenotype. These mutations are usually associated with chemoresistance and poor clinical outcomes [73, 74, 75]. Missense mutations within the DBD often result in the production of a full‐length, but highly stable protein that suppresses wild‐type p53 activity encoded by the second allele. These variants exhibit double‐negative (DN) effects that promote leukemogenesis and cellular survival. Notably, the majority of the DBD mutants exhibit DN activity [76, 77]. In addition to LOF effects, certain TP53 mutations confer gain‐of‐function (GOF) properties, promoting tumorigenesis, by inhibiting other tumor suppressor proteins, such as p63 and p73 [78, 79].

Mutations in the OD domain are relatively rare as acquired somatic events but are frequently observed as germline mutations [80]. These mutation alterations disrupt tetramerization and consequently impair DNA binding, generally resulting in LOF [80].

*TP53* mutations in AML are strongly associated with complex karyotype, chromothripsis, chromosomal arm losses (particularly involving chromosomes 5, 7, 17), and aneuploidy. Conversely, they are rarely linked to low‐risk cytogenetic aberrations such as t(8:21) or inv 16 [81]. Furthermore, *TP53* mutations seldom co‐occur with alterations in the *RAS* pathway (4%), *FLT3* (6%), *NPM1* (8%), or single‐nucleotide variants in recurrent AML‐associated genes such as (*DNMT3A, TET2, IDH1/2*) [81, 82]. Notably, the pattern of co‐mutations can differ between *TP53*‐mutated founder clones and subclones. Recent studies underscore the prognostic significance of variant allele frequency (VAF) in *TP53‐*mutated AML. *TP53* mutations are typically categorized into three VAF subgroups: (i) > 40%, (ii) 20%–40%, (iii) < 20%. Even at a VAF of < 20%, *TP53* mutations negatively impact overall survival (OS), event‐free survival (EFS), and complete remission (CR) rates [83]. Moreover, Sallman et al. demonstrated in myelodysplastic syndrome (MDS) that increasing *TP53* VAF correlates with worsening karyotype complexity and reduced OS [84].

In summary, *TP53* mutations in AML disrupt critical tumor‐suppressive functions, enabling leukemic cells to evade apoptosis, accumulate genomic instability, and undergo uncontrolled proliferation. These mutations are predominantly observed in secondary AML and are associated with disease aggressiveness and therapeutic resistance. Elucidating the molecular mechanisms underlying *TP53* mutations is essential for developing targeted therapeutic strategies to improve outcomes in *TP53*‐mutant AML.

## 4 Treatment for *TP53* Mutated AML

Unlike other AML subtypes that have seen significant therapeutic advancements, *TP53*‐mutated AML remains notoriously difficult to treat, with standard chemotherapy offering limited long‐term benefits. The management of *TP53*‐mutated AML is at a crossroads, with emerging therapies providing incremental progress but no definitive breakthroughs [85]. Table 1 summarizes treatment strategies in *TP53*‐mutated AML and high‐risk MDS.

**TABLE 1:** Clinical trial investigating conventional chemotherapy and novel agents in TP53‐mutated acute myeloid leukemia (AML) and high‐risk myelodysplastic syndromes (MDS).

Disease status	Phase study	Treatmen regimen	TP53mutated	Median OS in TP53 mutated (months)	cCR in TP53 mutated (%)	Reference
TN secondary AML	III	Experimental arm: CPX‐351	na	na	na	89
		Comparator arm: 3 + 7				
TN secondary AML	Retrospective	CPX‐351	18	na	33	90
TN AML ineligible for IC	II	DAC 5‐day	5‐day arm: 7	5‐day arm: 5.5	5‐day arm: 29	94
		DAC 10‐day	10‐day arm: 17	10‐day arm: 4.7	10‐day arm: 47	
TN AML ineligible for IC	III	Experimental arm: AZA‐VEN	Experimental arm: 54	Experimental arm: 5.2	Experimental arm: 40.7	100
		Comparator arm: AZA‐Placebo	Comparator arm: 18	Comparator arm: 4.9	Comparator arm: 16.7	
RR AML	II	Nivolumab and AZA	16	na	18.7	102
RR AML	II	Pembrolizumab and HIDAC	5	na	na	103
TN HR MDS or AML	II	Nivolumab, idarubicin, cytarabine	8	na	50	104
TN AML ineligible for IC	II	Experimental arm: Darvalumab and AZA	Experimental arm: 33	na	na	105
		Comparator arm: AZA	Comparator arm: 26			
TN AML	Ib	Magrolimab and AZA	72	9.8	40.3	110
TN HR MDS or AML	II	Eprenetapopt and AZA	52	HR MDS: 12.1	HR MDS: 62	119
				AML: 10.4	AML: 33	
TN AML	I	Entrectinib and ASTX727	13	na	43	142

Abbreviations: AML, acute myeloid leukemia; AZA, Azacitidine; cCR, composite complete remission; DAC, decitabine; HIDAC, high dose cytarabine; HR MDS, high risk myelodysplastic syndrome; IC, intensive chemotherapy; OS, overall survival; RR, relapsed or refractory; TN, treatment naïve.

### 4.1 Intensive Chemotherapy

For AML patients who are eligible for intensive chemotherapy, the current European LeukemiaNet (ELN) guidelines recommend induction therapy with a combination of anthracycline and cytarabine. The addition of gemtuzumab ozogamicin (GO) is advised for low‐ and intermediate‐risk patients, while midostaurin is incorporated for those harboring a *FLT3* mutation. However, for patients with *TP53*‐mutated AML, classified as high‐risk, standard frontline treatment consists of the 3 + 7 regimen (7 days of cytarabine and 3 days of an anthracycline such as daunorubicin or idarubicin) for de novo AML or CPX‐351, a liposomal formulation of cytarabine and daunorubicin, for secondary AML. Unfortunately, AML patients with *TP53* mutations tend to have a poor response to cytotoxic chemotherapy (28%–42%) and an OS of 5–9 months [6, 7, 8, 9]. Despite the widespread use of the standard 3 + 7 regimen, *TP53*‐mutated AML demonstrates a markedly poor response to cytotoxic agents, with reported CR rates ranging from 20% to 40% [86], underscoring the inherent chemoresistance conferred by *TP53* dysfunction.

Given the poor response to standard chemotherapy, CPX‐351 was developed as an alternative for patients with secondary AML, including those with therapy‐related disease and antecedent hematologic disorders. This dual‐drug encapsulated formulation, which preferentially delivers a synergistic 5:1 drug ratio of cytarabine to daunorubicin, has been shown to improve drug delivery to leukemic cells. A phase III clinical trial comparing CPX‐351 to the 3 + 7 regimen demonstrated significantly improved survival outcomes, particularly in patients with adverse‐risk cytogenetics. Treatment with CPX‐351 resulted in a median OS of 9.56 versus 5.95 months with 3 + 7, with higher CR rates (47.7% vs. 33.3%) and a comparable safety profile. No data are available about the prevalence of *TP53* mutations among patients enrolled in this study [87].

In a retrospective study conducted by Goldberg et al., which evaluated 101 patients treated with at least 1 cycle of CPX‐351, molecular profiling was available for 84 patients, among whom 21.4% carried TP53 mutations. Following 1‐2 cycles of induction therapy, the composite CR (cCR) rate was significantly higher in TP53 WT compared to TP53 mutant (62% vs. 33%). While OS was not significantly different between the two groups, there was a trend favoring improved survival in TP53 WT patients [88].

Further insights into the efficacy of CPX‐351 in high‐risk AML were provided by Othaman et al. in the UK NCRI AML19 trial, which compared CPX‐351 with FLAG‐Ida (fludarabine, cytarabine, granulocyte colony‐stimulating factor, and idarubicin) in younger adults with treatment‐naïve AML or high‐risk MDS. In this cohort, TP53 mutations were the most frequently detected genetic alteration, occurring in 43% of patients. Following two cycles of therapy, the overall response rates (ORR) of the entire population enrolled in the study were 64% with CPX‐351% and 76% with FLAG‐Ida, but there was no difference in OS (13.3 vs. 11.4 months) or EFS between treatment arms. However, relapse‐free survival (RFS) was significantly longer with CPX‐351 (median 22.1 vs. 8.35 months). Notably, no survival differences were observed between patients with secondary AML and those with MDS‐related cytogenetic abnormalities, suggesting that these entities share a similar disease trajectory and response to therapy [89].

### 4.2 Hypometilating Agents (HMAs)

Current clinical guidelines recommend azacitidine (AZA) and decitabine (DAC)‐based regimens as treatment options for patients ineligible for intensive chemotherapy [6]. Preclinical studies have suggested that p53‐deficient cells exhibit increased sensitivity to HMAs [90], indicating a potential therapeutic advantage in *TP53*‐mutated AML. This hypothesis was supported by a single‐center study involving 116 patients with AML/MDS harboring *TP53* mutations, who were treated with a 10‐day decitabine in a 28‐day cycle. Interestingly, response rates were higher among patients with an unfavorable‐risk cytogenetic profile compared to those with intermediate‐risk or favorable‐risk profiles (67% vs. 34%, *p* < 0.001). Furthermore, patients with *TP53* mutations demonstrated superior response rates compared to *TP53* WT (100% vs. 41%, *p* < 0.001). Overall, 15 of the 116 patients in the combined patient cohorts (13%) had a complete remission, and an additional 38 patients had bone marrow blast clearance with less than 5% blasts, resulting in an overall response rate of 46%. Nine patients (8%) experienced a partial response, 23 (20%) a stable disease, and 19 (16%) a progression. In this study, responses correlated with the median number of cycles and performance status, while no correlation was found with age or white‐cell counts, as with the steady‐state plasma decitabine levels on day 4 of the 1st cycle and the reduction of CpG methylation from day 0 to day 10 of the 1st cycle. Analyzing the spectrum of *TP53*‐mutations in responders, it resulted very similar to that of mutations reported in other studies of AML Considering the higher response rate reported in TP53 mutated patients in this study, a further analysis was performed to better understand if different type of *TP53* mutations could influence results founding no significant difference in clinical outcomes between these two schedules [91]. In a subsequent phase II study, Short et al. assessed the efficacy of a 5‐day versus a 10‐day DAC regimen in *TP53*‐mutated AML patients. Interestingly, response rates appeared to be independent of *TP53* VAF, suggesting that decitabine efficacy is not influenced by the mutational burden of TP53 [92].

### 4.3 Venetoclax‐Based Regimen

The introduction of the Bcl2 inhibitor venetoclax in combination with HMAs has significantly changed the treatment paradigm of AML patients ineligible for intensive chemotherapy. This combination has been shown to improve OS, increasing from 9.6 months with AZA alone to 14.7 months with the addition of venetoclax (AZA‐VEN) [93]. However, in the subset of patients harboring *TP53* mutations, the survival benefit was not statistically significant [93]. Several studies have reported higher response rates with the combination of HMAs and venetoclax compared to HMAs alone in *TP53*‐mutated AML, but this did not translate into a meaningful improvement in OS or RFS [94, 95, 96]. A retrospective analysis of 103 patients treated with venetoclax in combination with HMAs outside clinical trials further showed that the presence of *TP53* mutations was associated with shorter OS [97]. A post hoc analysis of VIALE‐A assessed 127 patients with poor‐risk cytogenetics features, including both *TP53* WT and mutated cases. Among patients with *TP53* WT and adverse cytogenetics, the addition of venetoclax to azacitidine resulted in a cCR of 70% versus 23%, a median duration of remission (DoR) of 18.4 versus 8.5 months, and a median OS of 23.4 versus 11.3 months. In contrast, for *TP53* mutated patients with poor‐risk cytogenetics, venetoclax addiction led to a cCR of 41% versus 17%, a median DoR of 6.5 versus 6.7 months, and a median OS of 5.2 versus 4.9 months [98].

These findings highlight the limited efficacy of venetoclax‐based regimens in *TP53*‐mutated AML.

### 4.4 Checkpoint Inhibitors

Following the success of immunotherapy in lymphoid malignancies, there has been growing interest in exploring its potential in AML. Among monoclonal antibodies, checkpoint inhibitors have emerged as promising agents due to their ability to block inhibitory coreceptors on T cells, including PD1, PD‐L1, and cytotoxic T‐lymphocyte antigen 4 inhibitors. Preclinical studies have demonstrated an increased expression of PD‐1 in *TP53*‐mutated AML cells, likely driven by MYC upregulation and miR‐34a downregulation. Additionally, AML is characterized by a reduction in cytotoxic and helper T cells, alongside an expansion of immunosuppressive regulatory T cells and myeloid‐derived suppressor cells, contributing to an immune‐suppressive microenvironment [99].

To enhance the immune response against leukemic cells, checkpoint inhibitors have been combined with HMAs or intensive chemotherapy. In a recent phase II study evaluating the combination of nivolumab and azacitidine in 70 relapsed/refractory (RR) AML patients, 16 harbored *TP53* mutations, among whom the ORR was 19% (3 responders) [100]. Another phase II study investigated the administration of pembrolizumab following high‐dose cytarabine in 37 patients with RR AML, including five with *TP53* mutations, and among those two achieved CR [101].

More recently, a phase II trial assessed the addition of nivolumab to idarubicin and cytarabine in 44 treatment‐naïve patients with AML or high‐risk MDS, including eight with *TP53*‐mutated MDS but not AML, reporting a cCR rate of 78% in the overall population [102]. Similarly, a phase II study evaluated the addition of the anti–PD‐L1 antibody durvalumab to azacitidine in first‐line treatment of high‐risk MDS or AML in patients unfit for intensive treatment, but no significant improvement in response rate or OS was observed [103, 104].

Collectively, these findings suggest that checkpoint inhibitors may not significantly improve survival in AML as monotherapy or in combination with HMAs.

### 4.5 Sabatolimab

Sabatolimab is a humanized monoclonal antibody targeting T‐cell immunoglobulin and mucin domain‐containing protein 3 (TIM‐3), a key immunoregulatory receptor involved in immune evasion [105]. A phase Ib study evaluated the safety and efficacy of sabatolimab in combination with HMAs in patients with AML, high‐risk MDS, or chronic myelomonocytic leukemia (CMML). The combination showed durable clinical responses observed in patients with high‐risk or very high‐risk MDS, including those harboring the *TP53* mutation. Similarly, in treatment‐naïve AML patients with at least one adverse‐risk mutation (*ASXl1, RUNX1,* or *TP53*), the ORR was 53.8% and a median DOR of 12.6 months [106].

The phase II STIMULUS‐AML‐1 trial investigated the combination of sabatolimab, venetoclax, and azacitidine in treatment‐naïve AML patients with a good safety profile [107].

### 4.6 Anti CD47 Monoclonal Antibodies

Anti‐CD47 monoclonal antibodies, such as magrolimab, represent a promising therapeutic approach in cancer immunotherapy. Magrolimab functions by inhibiting the interaction between CD47, an antiphagocytic glycoprotein expressed on tumor cells, and signal regulatory protein alpha (SIRPα) on macrophages, thereby enhancing phagocytosis and promoting tumor cell elimination. CD47 signals a “don't eat me” message to macrophages, thereby protecting tumor cells from immune surveillance [108]. In a Phase Ib trial, magrolimab, when combined with azacitidine, demonstrated promising results in 87 patients with TN AML, of whom 72 (82.8%) carried the *TP53* mutation. Among this cohort, the rate of cCR was 40.3%, with a CR rate of 31.9% [109]. Following these encouraging results, two Phase III trials were planned—ENHANCE‐2 and ENHANCE‐3—to further assess magrolimab's efficacy. The ENHANCE‐2 study compared magrolimab combined with azacitidine to the physician's choice of venetoclax with azacitidine or intensive chemotherapy in patients with *TP53*‐mutated TN AML, while ENHANCE‐3 evaluated magrolimab *versus* placebo in combination with venetoclax and azacitidine in TN AML. However, Gilead recently announced the discontinuation of the ENHANCE‐2 study, based on an interim analysis, which, following review by an independent data monitoring committee, concluded that Magrolimab is unlikely to confer a survival benefit over the standard of care in *TP53*‐mutated AML [110].

Despite this setback, other CD‐47‐targerting antibodies, such as evorpacept, continue to be explored. The Phase 1/2 ASPEN‐05 trial tested the safety and efficacy of evorpacet in combination with venetoclax and azacitidine in RR or TN AML patients with adverse‐risk genetics, who were ineligible for intensive induction therapy. A total of 18 patients were enrolled, including 11 with RR and 3 TN AML, of whom 10 had *TP53* mutations. All subjects experienced at least one AE, but the maximum tolerated dose was not reached. Objective responses were observed in 3 TN patients and 4 RR patients, including 2 venetoclax naïve and 2 venetoclax‐exposed patients [111].

### 4.7 Anti CD123 Monoclonal Antibodies

Tagraxofusp is a first‐in‐class monoclonal antibody targeting CD123, approved for the treatment of blastic plasmacytoid dendritic cell neoplasm. In a Phase 1b study, tagraxofusp was administered in combination with azacitidine in patients with CD123‐positive AML or high‐risk MDS, and later in combination with azacitidine and venetoclax in AML. In an expansion cohort of 26 patients with TN AML and adverse genetic features, including 13 patients with *TP53* mutations, the triplet combination of tagraxofusp, azacitidine, and venetoclax resulted in an ORR of 69%, with a CR rate of 39%. Seven of the 13 *TP53*‐mutated patients responded, with 4 achieving a CR. The median OS and PFS were 14 and 8.5 months, respectively. These data suggest that the addition of tagraxofusp may confer clinical benefit in *TP53*‐mutated patients with AML [112].

Flotetuzumab, a bispecific antibody targeting CD123 and CD3, has shown promising efficacy in RR *TP53*‐mutated AML. A preclinical study analyzed bone marrow samples from 64 patients with *TP53*‐mutated (42 cases) and TP53 WT AML (22 cases) as well as 45 BM samples from patients who received flotetuzumab for RR AML, including 15 cases with *TP53* mutations and/or 17p deletion. Bone marrow samples from *TP53*‐mutated patients exhibited higher expression of FOXP3, IFNG, markers of immune senescence, immune checkpoints, and phosphatidylinositol 3‐kinase‐Akt and NF‐κB signaling intermediates. Seven of 15 patients (47%) with RR AML and *TP53* abnormalities achieved CRs to flotetuzumab (< 5% BM blasts), with a significantly greater reduction in tumor inflammation signature, FOXP3, CD8, inflammatory chemokine, and PD1 gene expression compared to non‐responders [113].

### 4.8 Eprenetapopt

Eprenetapopt, also known as APR‐246, is a novel small drug that restores the normal p53 function in both mutated and wild‐type p53 cells. A preclinical study showed that APR‐246 showed dose‐dependent pro‐apoptotic and cytotoxic effects in both AML cell lines and in leukemic cells from AML patients. The study showed that APR‐246 increases intracellular p53 protein levels, enhances caspase‐3 activity, and alters the bax/bcl‐2 ratio, suggesting a mechanism of action that promotes cell death. Interestingly, pre‐incubation with APR‐246 enhanced the sensitivity of AML cells to conventional chemotherapeutic agents, including cytarabine, fludarabine, and above all, daunorubicin [114].

Moreover, a study by Ali et al. found that exposure to APR‐246 induces the formation of reactive oxygen species (ROS), depletes intracellular glutathione, and upregulates the expression of key genes, such as NFE2L2, HMOX1, RIT1, and SLC7A11. The PI3K inhibitor wortmannin and the mTOR inhibitor rapamycin were found to synergistically enhance cell killing via NF2L2 activation [115].

Additionally, APR‐246 may trigger ferroptosis in AML cells. The ability of AML cells to detoxify lipid peroxides by increasing their cystine uptake for glutathione biosynthesis appears to be a critical factor in determining the sensitivity to APR‐246. The potential of combining APR‐246 with ferroptosis or glutathione biosynthesis inhibitors represents an intriguing avenue for further exploration [116].

Given the promising preclinical results, a Phase II trial was conducted to evaluate the efficacy and safety of eprenetapopt in combination with azacitidine in TN AML or high‐risk MDS with *TP53* mutations. This study enrolled 52 patients (18 with AML and 34 with MDS). In the MDS cohort, the ORR was 62%, with a CR rate of 47%. In the AML cohort, the ORR was 33%, with a CR rate of 17%. Among responders, 73% achieved minimal residual disease negativity as assessed by next‐generation sequencing (NGS). The most common AEs were febrile neutropenia (36%) and neurologic AEs (40%), the latter being associated with older age and pre‐existing renal impairment. They resolved with temporary drug interruption without recurrence after adequate eprenetapopt dose reduction. After a median follow‐up of 9.7 months, the median OS was 12.1 months in MDS, and 13.9 months in AML with less than 30% marrow blasts, compared to 3.0 months for AML with more than 30% marrow blasts [117].

However, a subsequent Phase 3 trial combining eprenetapopt with azacitidine for *TP53*‐mutated MDS and AML did not meet its primary endpoint of improved CR rate. The CR rate was 33.3% in the experimental group compared to 22.4% in the control group (*p* = 0.13) [118]. These results may be influenced by the biological heterogeneity of *TP53* mutations, which can lead to varying responses across a diverse patient population [118].

### 4.9 MDM2 Inhibitors

MDM2 inhibitors have gained attention as a promising therapeutic strategy in AML due to their ability to restore p53 function by blocking the MDM2‐p53 axis [15, 16, 17, 18, 19]. MDM2 is frequently overexpressed in AML, promoting p53 degradation and contributing to oncogenesis. Preclinical studies have demonstrated the anti‐leukemic activity of MDM2 inhibitors, both as monotherapy and in combination with HMAs or cytarabine [119]. Several clinical studies have evaluated the efficacy of these drugs alone or in combination with chemotherapy. A phase I study assessed the efficacy and safety of milademetan, an MDM2 inhibitor, as monotherapy and in combination with azacitidine in RR AML or high‐risk MDS. A total of 57 patients received milademetan as monotherapy, and 17 received it in combination with azacitidine.

Dose‐limiting toxicities were gastrointestinal issues, fatigue, or renal/electrolyte abnormalities. AEs related to milademetan occurred in 82.5% of patients in the monotherapy arm and 64.7% of those in the combination group. Unfortunately, the CR rate was low, with only 2 patients (4.2%) achieving a CR in the monotherapy arm, and 1 (2.1%) achieving CR with incomplete blood count recovery (CRi). In the combination arm, 2 participants (13.3%) achieved CRi [120].

In another Phase 1b trial, the combination of idasanutlin, another MDM2 inhibitor, and venetoclax was tested in RR AML ineligible for IC. Common AEs included diarrhea (87.3%), nausea (74.5%), vomiting (52.7%), hypokalemia (50.9%), and febrile neutropenia (45.5%). Across all dose groups, the cCR rate was 26%, and the morphologic leukemia‐free state (MLFS) rate was 12%. For the recommended phase II doses (venetoclax 600 mg + idasanutlin 150 mg; venetoclax 600 mg + idasanutlin 200 mg), the combined CRc rate was 34.3% and the MLFS rate was 14.3%. In patients with *TP53*‐mutated AML, the cCR rate was 20%, with responses observed in those with co‐occurring IDH and RUNX1 mutations. Notably, 25 emergent *TP53* mutations were detected in 12 of 36 evaluable patients, with 22 of these mutations present at baseline with low *TP53* variant allele frequency [121].

The Phase III MIRROS trial explored the efficacy of idasanutlin in combination with cytarabine in RR ANML patients. A total of 447 patients were enrolled, regardless of *TP53* mutation status, and randomly assigned to receive idasanutlin 300 mg orally twice daily or placebo, along with cytarabine 1 g/m^2^ IV on days 1–5 of 28‐day cycles. At primary analysis, 436 patients were analyzed, including 355 in the *TP53* wild‐type intention‐to‐treat (*TP53*WT‐ITT) population. In the *TP53*WT‐ITT group, the median OS was 8.3 months in the idasanutlin‐cytarabine group compared to 9.1 months in the placebo‐cytarabine group (*p* = 0.58). The CR rate was 20.3% for the idasanutlin‐cytarabine combination and 17.1% for placebo‐cytarabine. No data on the *TP53*‐mutated population or the emergence of *TP53* mutations during treatment have been reported [122].

### 4.10 Allogenic Stem Cell Transplantation (alloSCT)

AlloSCT is considered the preferred consolidation treatment for high‐risk AML when applicable, according to clinical guidelines [6]. However, patients with *TP53* mutations exhibit a poor prognosis even after undergoing alloSCT.

A multicenter study involving 68 patients with *TP53*‐mutated AML who underwent alloSCT showed a median EFS of 12.4 months and a median OS of 24.5 months. The incidence of acute graft versus host disease (GVHD) was 37%, and chronic GVHD was 44%. Multivariate analysis revealed that CR at day 100 post alloSCT and chronic GVHD were significant factors for both EFS and OS [123].

In a retrospective analysis conducted at MD Anderson Cancer Center, 83 AML patients with *TP53*‐mutated AML were analyzed, showing a median OS and PFS of 8 and 5 months, respectively. The 1‐year non‐relapse mortality (NRM) rate was 20%, while the relapse rate was 53%. They stratified patients based on the Karnofsky performance status. Factors associated with a dismal prognosis were HCT‐CI > 4, Karnofsky performance status ≤ 80%, and residual leukemia at the time of transplant [124].

The European Society for Blood and Marrow Transplantation (EBMT) reported a significantly lower 2‐year OS in patients with *TP53*‐mutated AML compared to those with *TP53* WT AML, at 35.1 *versus* 64%, respectively. However, when patients with either chromosome 17p loss (17p‐) or complex karyotype were excluded, the 2‐year OS was 65.2%, which was comparable to those with *TP53* WT AML [125]. Given the wide range of *TP53* mutations, the impact of different types of mutations on post‐alloSCT outcomes remains unclear. Linsday et al. suggested that patients with truncating *TP53* variants may have a worse prognosis, but the role of specific *TP53* variant types in post‐alloSCT outcomes requires further investigation [126].

Another critical consideration is the high risk of relapse in high‐risk AML, even after alloSCT. Several maintenance strategies have been explored to address this challenge, most with unsatisfactory results [127].

However, a Phase II study testing the combination of azacitidine and eprenetapopt as maintenance therapy showed promising results. After a median follow‐up of 14.5 months, the median RFS was 12.5 months, and after a median follow‐up of 17 months, the median OS was 20.6 months, with an acceptable toxicity profile [128].

## 5 Discussion

*TP53*‐mutated AML is one of the most challenging hematologic malignancies to treat, with a consistently poor prognosis despite multiple therapeutic advancements. The aggressive nature of this disease is largely attributed to the central role of *TP53* in genomic stability, apoptosis, and DNA repair. Unlike other AML subtypes, where targeted therapies have improved outcomes, *TP53*‐mutated AML remains largely resistant to conventional and novel approaches alike. Over the years, various strategies have been tested, yet most have failed to deliver meaningful long‐term benefits. Understanding why these therapies have fallen short is critical for paving the way for more effective treatment paradigms.

Why Have Therapies Failed?

The first‐line treatment for AML in eligible patients has traditionally been IC. However, *TP53* mutations disrupt the very mechanisms that chemotherapy depends on, namely, DNA damage‐induced apoptosis. As a result, responses to IC are often short‐lived, with most patients relapsing within a few months. Moreover, allo‐SCT, which is considered curative in other AML subtypes, has shown disappointing results in *TP53*‐mutated patients, with relapse rates exceeding 80% and minimal survival benefit [6, 86].

The advent of HMAs like AZA and DAC initially brought some hope for *TP53*‐mutated AML, particularly with extended decitabine dosing schedules. Preclinical data suggested that *TP53*‐deficient cells might be particularly sensitive to HMAs, and early studies showed promising response rates. However, the survival advantage was marginal, with most responses being transient. Even the introduction of venetoclax, a BCL‐2 inhibitor that revolutionized AML treatment in older patients, failed to yield a significant benefit in the *TP53*‐mutated subset. While response rates improved when venetoclax was added to HMAs, survival outcomes remained largely unchanged, likely due to the activation of alternative anti‐apoptotic pathways such as MCL‐1 and BCL‐XL [90, 91, 92, 93, 94, 95, 96, 97, 98].

Recent meta‐analyses have assessed the outcomes of IC and HMAs, with or without venetoclax in treatment‐naïve *TP53*‐mutated AML. These studies reaffirm that *TP53*‐mutated AML is associated with poor outcomes across different treatment strategies, with CR rates ranging from 13% to 43%. Interestingly, IC showed the highest CR/CRi rates (46% and 43%, respectively), though this finding is confounded by the tendency for IC protocols to enroll younger, fitter patients more likely to progress to alloSCT. Median overall survival (OS) estimates for each treatment type were uniformly low, ranging from a negligible 6.1–6.5 months for HMA‐based treatments and IC [86, 129].

Checkpoint inhibitors, which have transformed the treatment of many solid and hematologic malignancies, have also failed to make a meaningful impact in *TP53*‐mutated AML. Clinical trials of PD‐1 and PD‐L1 inhibitors, whether combined with chemotherapy or HMAs, reported only modest response rates and no significant survival advantage [100, 101, 102, 103, 104].

Similarly, sabatolimab, a TIM‐3 inhibitor designed to enhance immune responses against AML, showed limited efficacy when combined with HMAs. Although it had a favorable safety profile, response rates in *TP53*‐mutated patients were underwhelming [105, 106, 107]. Altogether, these data underscore the difficulty of targeting an immune system that is already profoundly dysregulated in AML.

Expanding into another area, Cheng et al. demonstrated that arsenic trioxide can restore p53 function by targeting an allosteric cryptic site. When combined with low‐dose cytarabine for treatment‐naïve AML or acute promyelocytic leukemia (APL), it achieved a CR rate of 34%, including 30% in patients with secondary or therapy‐related AML and adverse cytogenetics. However, this study did not assess *TP53* mutation status, limiting its applicability to *TP53*‐mutated [130, 131]. In this respect, a Phase II study is evaluating oral arsenic trioxide combined with low‐intensity treatment for TN or RR *TP53*‐mutated myeloid malignancies (NCT06778187).

Additional research explores two potential strategies for rescuing truncated p53: (i) restoring full‐length p53 protein expression using drugs such as G418 and NB124, which promote full‐length p53 production [132, 133] and (ii) inhibiting nonsense‐mediated decay (NMD) [134]. These approaches remain in the preclinical evaluation stage. Additionally, some *TP53* mutations lead to the formation of alternative p53 isoforms with oncogenic properties, driving cancer cell proliferation and survival. In such cases, rescue therapies may be ineffective, and targeted degradation strategies could be more beneficial [135, 136].

Preclinical studies have also highlighted the efficacy of non‐cancer‐specific drugs that modulate various gene targets [137, 138]. Given the potential of multitarget strategies in overcoming resistance, ongoing trials are investigating the combination of nivolumab, decitabine, and venetoclax in treatment‐naïve *TP53*‐mutated AML (NCT04277442).

Given these challenges, what are the next steps?

The failure of these treatments does not mean that *TP53*‐mutated AML is untreatable—it simply highlights the need for smarter, multi‐faceted approaches. One avenue that remains promising is the reactivation of mutant p53 itself. APR‐246 (eprenetapopt) was developed with this goal in mind, showing encouraging early results when combined with azacitidine. However, its phase III trial failed to demonstrate a clear survival benefit, suggesting that patient selection or combination strategies need to be refined [118]. More research is needed to identify which *TP53* mutations are truly susceptible to reactivation therapies.

Recent Phase 1 studies have explored novel combinations, such as entrectinib, a neurotrophin receptor kinase (NTRK) inhibitor used in solid tumors, with oral decitabine and cedazuridine (ASTX727) in RR AML with *TP53* mutations. Of the 13 patients enrolled, one experienced dose‐limiting toxicity (DLT), and three patients (43%) had stable disease after the first cycle. One patient (14%) achieved a CR lasting 5 months [139]. Another Phase I study is investigating atorvastatin in both *TP53*‐mutated and *TP53* wild‐type malignancies (NCT03560882).

A key focus is simultaneously inhibiting multiple survival pathways. Since *TP53*‐mutated cells can bypass apoptosis through non‐BCL‐2 mechanisms, combining venetoclax with MCL‐1 or CHK1 inhibitors may improve responses. Early‐phase trials of such dual‐inhibition strategies are currently underway [140, 141].

The potential of immunotherapy remains underexplored. The failure of checkpoint inhibitors does not mean that all immune‐based strategies are futile. Bispecific T‐cell engagers (BiTEs) and CAR‐T cell therapies targeting AML antigens like CD123 and CD33 offer a different approach that bypasses some of the limitations of checkpoint blockade. These therapies are still in their early stages, but if the immunosuppressive microenvironment can be effectively countered, they may offer new hope [142].

Finally, the bone marrow microenvironment itself is an underappreciated factor in *TP53*‐mutated AML resistance. Leukemic cells in this subtype may derive survival signals from their surrounding stromal cells, making them more resilient to treatment. Agents targeting the CXCR4 pathway or other bone marrow niche interactions could help sensitize *TP53*‐mutated AML cells to existing therapies [143].

## 6 Conclusion

The story of *TP53*‐mutated AML is one of persistent frustration but also ongoing scientific evolution. Each failed therapy has provided crucial insights into the biology of this disease, bringing us closer to understanding what truly drives resistance. The next generation of therapies will need to go beyond single‐agent approaches, integrating multiple targeted strategies to address the diverse survival mechanisms of *TP53*‐mutated cells. While challenges remain, rapid advancements in cancer biology, immunotherapy, and precision medicine may lead to improved outcomes in the near future.

## Author Contributions

All authors contributed to the manuscript and were involved in revisions and proofreading. All authors approved the submitted version.

## Conflicts of Interest

The authors declare no conflicts of interest.

### Peer Review

The peer review history for this article is available at https://www.webofscience.com/api/gateway/wos/peer-review/10.1002/hon.70106.

## Data Availability

Data sharing is not applicable to this article as no new data were created or analyzed in this study.

## References

[1] M. Sant , C. Allemani , C. Tereanu , et al., and HAEMACARE Working Group , “Incidence of Hematologic Malignancies in Europe by Morphologic Subtype: Results of the HAEMACARE Project,” Blood 116, no. 19 (November 2010): 3724–3734: Epub 2010 Jul 27. Erratum in: Blood. 2011 Mar 24;117(12):3477. PMID: 20664057, 10.1182/blood-2010-05-282632. 20664057

[2] G. M. Dores , S. S. Devesa , R. E. Curtis , M. S. Linet , and L. M. Morton , “Acute Leukemia Incidence and Patient Survival Among Children and Adults in the United States, 2001‐2007,” Blood 119, no. 1 (January 2012): 34–43: Epub 2011 Nov 15. PMID: 22086414; PMCID: PMC3251235, 10.1182/blood-2011-04-347872. 22086414 PMC3251235

[3] D. A. Arber , A. Orazi , R. P. Hasserjian , et al., “International Consensus Classification of Myeloid Neoplasms and Acute Leukemias: Integrating Morphologic, Clinical, and Genomic Data,” Blood 140, no. 11 (September 2022): 1200–1228: PMID: 35767897; PMCID: PMC9479031, 10.1182/blood.2022015850. 35767897 PMC9479031

[4] J. D. Khoury , E. Solary , O. Abla , et al., “The 5th Edition of the World Health Organization Classification of Haematolymphoid Tumours: Myeloid and Histiocytic/Dendritic Neoplasms,” Leukemia 36, no. 7 (July 2022): 1703–1719: Epub 2022 Jun 22. PMID: 35732831; PMCID: PMC9252913, 10.1038/s41375-022-01613-1. 35732831 PMC9252913

[5] National Cancer Institute . Surveillance, Epidemiology, and End Results Program (SEER). Cancer Stat Facts: Leukemia ‐ Acute Myeloid Leukemia (AML). Updated 2022, https://seer.cancer.gov/statfacts/html/amyl.html.

[6] H. Döhner , A. H. Wei , F. R. Appelbaum , et al., “Diagnosis and Management of AML in Adults: 2022 Recommendations From an International Expert Panel on Behalf of the ELN,” Blood 140, no. 12 (September 2022): 1345–1377: PMID: 35797463, 10.1182/blood.2022016867. 35797463

[7] C. Kandoth , M. D. McLellan , F. Vandin , et al., “Mutational Landscape and Significance Across 12 Major Cancer Types,” Nature 502, no. 7471 (October 2013): 333–339: PMID: 24132290; PMCID: PMC3927368, 10.1038/nature12634. 24132290 PMC3927368

[8] A. C. Joerger and A. R. Fersht , “The Tumor Suppressor p53: From Structures to Drug Discovery,” Cold Spring Harbor Perspectives in Biology 2, no. 6 (June 2010): a000919: Epub 2010 Feb 10. PMID: 20516128; PMCID: PMC2869527, 10.1101/cshperspect.a000919. 20516128 PMC2869527

[9] E. Papaemmanuil , M. Gerstung , L. Bullinger , et al., “Genomic Classification and Prognosis in Acute Myeloid Leukemia,” New England Journal of Medicine 374, no. 23 (June 2016): 2209–2221: PMID: 27276561; PMCID: PMC4979995, 10.1056/NEJMoa1516192. 27276561 PMC4979995

[10] A. Stengel , W. Kern , T. Haferlach , M. Meggendorfer , A. Fasan , and C. Haferlach , “The Impact of TP53 Mutations and TP53 Deletions on Survival Varies Between AML, ALL, MDS and CLL: An Analysis of 3307 Cases,” Leukemia 31, no. 3 (March 2017): 705–711: Epub 2016 Sep 29. PMID: 27680515, 10.1038/leu.2016.263. 27680515

[11] D. Hiwase , C. Hahn , E. N. H. Tran , et al., “TP53 Mutation in Therapy‐Related Myeloid Neoplasm Defines a Distinct Molecular Subtype,” Blood 141, no. 9 (March 2023): 1087–1091: PMID: 36574363, 10.1182/blood.2022018236. 36574363

[12] T. Haferlach , “Advancing Leukemia Diagnostics: Role of Next Generation Sequencing (NGS) in Acute Myeloid Leukemia,” supplement, Hematology Reports 12, no. S1 (September 2020): 8957: PMID: 33042506; PMCID: PMC7520852, 10.4081/hr.2020.8957. 33042506 PMC7520852

[13] Y. Cho , S. Gorina , P. D. Jeffrey , and N. P. Pavletich , “Crystal Structure of a p53 Tumor Suppressor‐DNA Complex: Understanding Tumorigenic Mutations,” Science 265, no. 5170 (July 1994): 346–355: PMID: 8023157, 10.1126/science.8023157. 8023157

[14] A. N. Bullock and A. R. Fersht , “Rescuing the Function of Mutant p53,” Nature Reviews Cancer 1, no. 1 (October 2001): 68–76: PMID: 11900253, 10.1038/35094077. 11900253

[15] J. Momand , H. H. Wu , and G. Dasgupta , “MDM2‐‐Master Regulator of the p53 Tumor Suppressor Protein,” Gene 242, no. 1–2 (January 2000): 15–29: PMID: 10721693, 10.1016/s0378-1119(99)00487-4. 10721693

[16] K. T. Bieging , S. S. Mello , and L. D. Attardi , “Unravelling Mechanisms of p53‐Mediated Tumour Suppression,” Nature Reviews Cancer 14, no. 5 (May 2014): 359–370: Epub 2014 Apr 17. PMID: 24739573; PMCID: PMC4049238, 10.1038/nrc3711. PMC4049238 24739573

[17] A. Sigal and V. Rotter , “Oncogenic Mutations of the p53 Tumor Suppressor: The Demons of the Guardian of the Genome,” Cancer Research 60, no. 24 (December 2000): 6788–6793: PMID: 11156366. 11156366

[18] P. N. Friedman , X. Chen , J. Bargonetti , and C. Prives , “The p53 Protein is an Unusually Shaped Tetramer That Binds Directly to DNA,” Proceedings of the National Academy of Sciences 90, no. 8 (April 1993): 3319–3323: Erratum in: Proc Natl Acad Sci U S A 1993 Jun 15;90(12):5878. PMID: 8475074; PMCID: PMC46291, 10.1073/pnas.90.8.3319. PMC46291 8475074

[19] A. M. Bode and Z. Dong , “Post‐Translational Modification of p53 in Tumorigenesis,” Nature Reviews Cancer 4, no. 10 (October 2004): 793–805: PMID: 15510160, 10.1038/nrc1455. 15510160

[20] S. W. Lowe and A. W. Lin , “Apoptosis in Cancer,” Carcinogenesis 21, no. 3 (March 2000): 485–495: PMID: 10688869, 10.1093/carcin/21.3.485. 10688869

[21] C. J. Sherr and J. D. Weber , “The ARF/p53 Pathway,” Current Opinion in Genetics & Development 10, no. 1 (February 2000): 94–99: PMID: 10679383, 10.1016/s0959-437x(99)00038-6. 10679383

[22] A. M. Carr , “Cell Cycle: Piecing Together the p53 Puzzle,” Science 287, no. 5459 (March 2000): 1765–1766: PMID: 10755928, 10.1126/science.287.5459.1765. 10755928

[23] B. Vogelstein , D. Lane , and A. J. Levine , “Surfing the p53 Network,” Nature 408, no. 6810 (November 2000): 307–310: PMID: 11099028, 10.1038/35042675. 11099028

[24] S. Adimoolam and J. M. Ford , “p53 and DNA Damage‐Inducible Expression of the Xeroderma Pigmentosum Group C Gene,” Proceedings of the National Academy of Sciences 99, no. 20 (October 2002): 12985–12990: Epub 2002 Sep 19. PMID: 12242345; PMCID: PMC130573, 10.1073/pnas.202485699. PMC130573 12242345

[25] A. M. Narasimha , M. Kaulich , G. S. Shapiro , Y. J. Choi , P. Sicinski , and S. F. Dowdy , “Cyclin D Activates the Rb Tumor Suppressor by Mono‐Phosphorylation,” eLife 3 (June 2014): e02872: PMID: 24876129; PMCID: PMC4076869, 10.7554/eLife.02872. 24876129 PMC4076869

[26] O. W. Prall , B. Sarcevic , E. A. Musgrove , C. K. Watts , and R. L. Sutherland , “Estrogen‐Induced Activation of Cdk4 and Cdk2 During G1‐S Phase Progression is Accompanied by Increased Cyclin D1 Expression and Decreased Cyclin‐Dependent Kinase Inhibitor Association With Cyclin E‐Cdk2,” Journal of Biological Chemistry 272, no. 16 (April 1997): 10882–10894: PMID: 9099745, 10.1074/jbc.272.16.10882. 9099745

[27] J. W. Harper , G. R. Adami , N. Wei , K. Keyomarsi , and S. J. Elledge , “The p21 Cdk‐Interacting Protein Cip1 is a Potent Inhibitor of G1 Cyclin‐Dependent Kinases,” Cell 75, no. 4 (November 1993): 805–816: PMID: 8242751, 10.1016/0092-8674(93)90499-g. 8242751

[28] O. Stevaux and N. J. Dyson , “A Revised Picture of the E2F Transcriptional Network and RB Function,” Current Opinion in Cell Biology 14, no. 6 (December 2002): 684–691: PMID: 12473340, 10.1016/s0955-0674(02)00388-5. 12473340

[29] M. Fischer , M. Quaas , L. Steiner , and K. Engeland , “The p53‐p21‐DREAM‐CDE/CHR Pathway Regulates G2/M Cell Cycle Genes,” Nucleic Acids Research 44, no. 1 (January 2016): 164–174: Epub 2015 Sep 17. PMID: 26384566; PMCID: PMC4705690, 10.1093/nar/gkv927. 26384566 PMC4705690

[30] W. R. Taylor and G. R. Stark , “Regulation of the G2/M Transition by p53,” Oncogene 20, no. 15 (April 2001): 1803–1815: PMID: 11313928, 10.1038/sj.onc.1204252. 11313928

[31] E. Oda , R. Ohki , H. Murasawa , et al., “Noxa, a BH3‐Only Member of the Bcl‐2 Family and Candidate Mediator of p53‐Induced Apoptosis,” Science 288, no. 5468 (May 2000): 1053–1058: PMID: 10807576, 10.1126/science.288.5468.1053. 10807576

[32] K. Nakano and K. H. Vousden , “PUMA, a Novel Proapoptotic Gene, is Induced by p53,” Molecular Cell 7, no. 3 (March 2001): 683–694: PMID: 11463392, 10.1016/s1097-2765(01)00214-3. 11463392

[33] Y. Chen , X. Zhang , A. C. Dantas Machado , et al., “Structure of p53 Binding to the BAX Response Element Reveals DNA Unwinding and Compression to Accommodate Base‐Pair Insertion,” Nucleic Acids Research 41, no. 17 (September 2013): 8368–8376: Epub 2013 Jul 8. PMID: 23836939; PMCID: PMC3783167, 10.1093/nar/gkt584. 23836939 PMC3783167

[34] H. Wei , L. Qu , S. Dai , et al., “Structural Insight Into the Molecular Mechanism of p53‐Mediated Mitochondrial Apoptosis,” Nature Communications 12, no. 1 (April 2021): 2280: PMID: 33863900; PMCID: PMC8052441, 10.1038/s41467-021-22655-6. PMC8052441 33863900

[35] Leu J. I. , Dumont P. , Hafey M. , Murphy M. E. , and George D. L. , “Mitochondrial p53 Activates Bak and Causes Disruption of a Bak‐Mcl1 Complex,” Nature Cell Biology 6, no. 5 (May 2004): 443–450: Epub 2004 Apr 11. PMID: 15077116.+, 10.1038/ncb1123. 15077116

[36] G. T. Bommer , I. Gerin , Y. Feng , et al., “p53‐Mediated Activation of miRNA34 Candidate Tumor‐Suppressor Genes,” Current Biology 17, no. 15 (August 2007): 1298–1307: Epub 2007 Jul 26. PMID: 17656095, 10.1016/j.cub.2007.06.068. 17656095

[37] V. Tarasov , P. Jung , B. Verdoodt , et al., “Differential Regulation of microRNAs by p53 Revealed by Massively Parallel Sequencing: miR‐34a is a p53 Target That Induces Apoptosis and G1‐Arrest,” Cell Cycle 6, no. 13 (July 2007): 1586–1593: Epub 2007 May 11. PMID: 17554199, 10.4161/cc.6.13.4436. 17554199

[38] G. S. Wu , K. Kim , and W. S. el‐Deiry , “KILLER/DR5, a Novel DNA‐Damage Inducible Death Receptor Gene, Links the p53‐Tumor Suppressor to Caspase Activation and Apoptotic Death,” Advances in Experimental Medicine & Biology 465 (2000): 143–151: PMID: 10810622, 10.1007/0-306-46817-4_13. 10810622

[39] M. Müller , S. Wilder , D. Bannasch , et al., “p53 Activates the CD95 (APO‐1/Fas) Gene in Response to DNA Damage by Anticancer Drugs,” Journal of Experimental Medicine 188, no. 11 (December 1998): 2033–2045: PMID: 9841917; PMCID: PMC2212386, 10.1084/jem.188.11.2033. 9841917 PMC2212386

[40] B. Tiwari , A. E. Jones , and J. M. Abrams , “Transposons, p53 and Genome Security,” Trends in Genetics 34, no. 11 (November 2018): 846–855: Epub 2018 Sep 5. PMID: 30195581; PMCID: PMC6260979, 10.1016/j.tig.2018.08.003. 30195581 PMC6260979

[41] A. Haoudi , O. J. Semmes , J. M. Mason , and R. E. Cannon , “Retrotransposition‐Competent Human LINE‐1 Induces Apoptosis in Cancer Cells With Intact p53,” Journal of Biomedicine and Biotechnology 2004, no. 4 (2004): 185–194: PMID: 15467158; PMCID: PMC555774, 10.1155/S1110724304403131. 15467158 PMC555774

[42] S. Matoba , J. G. Kang , W. D. Patino , et al., “p53 Regulates Mitochondrial Respiration,” Science 312, no. 5780 (June 2006): 1650–1653: Epub 2006 May 25. PMID: 16728594, 10.1126/science.1126863. 16728594

[43] K. Bensaad , A. Tsuruta , M. A. Selak , et al., “TIGAR, a p53‐Inducible Regulator of Glycolysis and Apoptosis,” Cell 126, no. 1 (July 2006): 107–120: PMID: 16839880, 10.1016/j.cell.2006.05.036. 16839880

[44] C. Zhang , M. Lin , R. Wu , et al., “Parkin, a p53 Target Gene, Mediates the Role of p53 in Glucose Metabolism and the Warburg Effect,” Proceedings of the National Academy of Sciences 108, no. 39 (September 2011): 16259–16264: Epub 2011 Sep 19. PMID: 21930938; PMCID: PMC3182683, 10.1073/pnas.1113884108. PMC3182683 21930938

[45] P. Jiang , W. Du , X. Wang , et al., “p53 Regulates Biosynthesis Through Direct Inactivation of Glucose‐6‐Phosphate Dehydrogenase,” Nature Cell Biology 13, no. 3 (March 2011): 310–316: Epub 2011 Feb 20. PMID: 21336310; PMCID: PMC3110666, 10.1038/ncb2172. 21336310 PMC3110666

[46] F. Schwartzenberg‐Bar‐Yoseph , M. Armoni , and E. Karnieli , “The Tumor Suppressor p53 Down‐Regulates Glucose Transporters GLUT1 and GLUT4 Gene Expression,” Cancer Research 64, no. 7 (April 2004): 2627–2633: PMID: 15059920, 10.1158/0008-5472.can-03-0846. 15059920

[47] S. H. Moon , C. H. Huang , S. L. Houlihan , et al., “p53 Represses the Mevalonate Pathway to Mediate Tumor Suppression,” Cell 176, no. 3 (January 2019): 564–580.e19: Epub 2018 Dec 20. PMID: 30580964; PMCID: PMC6483089, 10.1016/j.cell.2018.11.011. 30580964 PMC6483089

[48] Y. Zhu , L. Gu , X. Lin , et al., “P53 Deficiency Affects Cholesterol Esterification to Exacerbate Hepatocarcinogenesis,” Hepatology 77, no. 5 (May 2023): 1499–1511: Epub 2023 Apr 17. PMID: 35398929; PMCID: PMC11186660, 10.1002/hep.32518. 35398929 PMC11186660

[49] L. Li , Y. Mao , L. Zhao , et al., “p53 Regulation of Ammonia Metabolism Through Urea Cycle Controls Polyamine Biosynthesis,” Nature 567, no. 7747 (March 2019): 253–256: Epub 2019 Mar 6. Erratum in: Nature. 2019 May;569(7758):E10. doi: 10.1038/s41586‐019‐1121‐7. PMID: 30842655, 30842655 10.1038/s41586-019-0996-7

[50] S. J. Wang , D. Li , Y. Ou , et al., “Acetylation is Crucial for P53‐Mediated Ferroptosis and Tumor Suppression,” Cell Reports 17, no. 2 (October 2016): 366–373: PMID: 27705786; PMCID: PMC5227654, 10.1016/j.celrep.2016.09.022. 27705786 PMC5227654

[51] Y. Liu and W. Gu , “p53 in Ferroptosis Regulation: The New Weapon for the Old Guardian,” Cell Death & Differentiation 29, no. 5 (May 2022): 895–910: Epub 2022 Jan 27. PMID: 35087226; PMCID: PMC9091200, 10.1038/s41418-022-00943-y. 35087226 PMC9091200

[52] T. Li , N. Kon , L. Jiang , et al., “Tumor Suppression in the Absence of p53‐Mediated Cell‐Cycle Arrest, Apoptosis, and Senescence,” Cell 149, no. 6 (June 2012): 1269–1283: PMID: 22682249; PMCID: PMC3688046, 10.1016/j.cell.2012.04.026. 22682249 PMC3688046

[53] Y. Wang , L. Yang , X. Zhang , et al., “Epigenetic Regulation of Ferroptosis by H2B Monoubiquitination and p53,” EMBO Reports 20, no. 7 (July 2019): e47563: Epub 2019 May 22. PMID: 31267712; PMCID: PMC6607012, 10.15252/embr.201847563. 31267712 PMC6607012

[54] Y. Ou , S. J. Wang , D. Li , B. Chu , and W. Gu , “Activation of SAT1 Engages Polyamine Metabolism With p53‐Mediated Ferroptotic Responses,” Proceedings of the National Academy of Sciences 113, no. 44 (November 2016): E6806–E6812: Epub 2016 Oct 3. PMID: 27698118; PMCID: PMC5098629, 10.1073/pnas.1607152113. PMC5098629 27698118

[55] B. Chu , N. Kon , D. Chen , et al., “ALOX12 is Required for p53‐Mediated Tumour Suppression Through a Distinct Ferroptosis Pathway,” Nature Cell Biology 21, no. 5 (May 2019): 579–591: Epub 2019 Apr 8. PMID: 30962574; PMCID: PMC6624840, 10.1038/s41556-019-0305-6. 30962574 PMC6624840

[56] J. Liu , C. Zhang , J. Wang , W. Hu , and Z. Feng , “The Regulation of Ferroptosis by Tumor Suppressor p53 and its Pathway,” International Journal of Molecular Sciences 21, no. 21 (November 2020): 8387: PMID: 33182266; PMCID: PMC7664917, 10.3390/ijms21218387. 33182266 PMC7664917

[57] Y. Xie , S. Zhu , X. Song , et al., “The Tumor Suppressor p53 Limits Ferroptosis by Blocking DPP4 Activity,” Cell Reports 20, no. 7 (August 2017): 1692–1704: PMID: 28813679, 10.1016/j.celrep.2017.07.055. 28813679

[58] B. D. Kenzelmann , M. S. Spano , K. T. Bieging , et al., “Global Genomic Profiling Reveals an Extensive p53‐Regulated Autophagy Program Contributing to Key p53 Responses,” Genes & Development 27, no. 9 (May 2013): 1016–1031: PMID: 23651856; PMCID: PMC3656320, 10.1101/gad.212282.112. 23651856 PMC3656320

[59] W. Zheng , Q. Chen , C. Wang , et al., “Inhibition of Cathepsin D (CTSD) Enhances Radiosensitivity of Glioblastoma Cells by Attenuating Autophagy,” Molecular Carcinogenesis 59, no. 6 (June 2020): 651–660: Epub 2020 Apr 6. PMID: 32253787, 10.1002/mc.23194. 32253787

[60] Y. J. Liu , T. Zhang , S. Chen , et al., “The Noncanonical Role of the Protease Cathepsin D as a Cofilin Phosphatase,” Cell Research 31, no. 7 (July 2021): 801–813: Epub 2021 Jan 29. PMID: 33514914; PMCID: PMC8249557, 10.1038/s41422-020-00454-w. 33514914 PMC8249557

[61] G. S. Wu , P. Saftig , C. Peters , and W. S. El‐Deiry , “Potential Role for Cathepsin D in p53‐dependent Tumor Suppression and Chemosensitivity,” Oncogene 16, no. 17 (April 1998): 2177–2183: Erratum in: Oncogene. 2025 Mar;44(9):630‐631. doi: 10.1038/s41388‐025‐03291‐6. PMID: 9619826, 9619826 10.1038/sj.onc.1201755

[62] S. Y. Yeo , Y. Itahana , A. K. Guo , et al., “Transglutaminase 2 Contributes to a TP53‐Induced Autophagy Program to Prevent Oncogenic Transformation,” eLife 5 (March 2016): e07101: PMID: 26956429; PMCID: PMC4798945, 10.7554/eLife.07101. 26956429 PMC4798945

[63] J. Kim , M. Kundu , B. Viollet , and K. L. Guan , “AMPK and mTOR Regulate Autophagy Through Direct Phosphorylation of Ulk1,” Nature Cell Biology 13, no. 2 (February 2011): 132–141: Epub 2011 Jan 23. PMID: 21258367; PMCID: PMC3987946, 21258367 10.1038/ncb2152 PMC3987946

[64] ÁF. Fernández , S. Sebti , Y. Wei , et al., “Disruption of the Beclin 1‐BCL2 Autophagy Regulatory Complex Promotes Longevity in Mice,” Nature 558, no. 7708 (June 2018): 136–140: Epub 2018 May 30. Erratum in: Nature. 2018 September;561(7723):E30. doi: 10.1038/s41586‐018‐0270‐4. PMID: 29849149; PMCID: PMC5992097, 29849149 10.1038/s41586-018-0162-7 PMC5992097

[65] E. F. Lee , N. A. Smith , T. P. Soares da Costa , et al., “Structural Insights Into BCL2 Pro‐Survival Protein Interactions With the Key Autophagy Regulator BECN1 Following Phosphorylation by STK4/MST1,” Autophagy 15, no. 5 (May 2019): 785–795: Epub 2019 Jan 9. PMID: 30626284; PMCID: PMC6526822, 10.1080/15548627.2018.1564557. 30626284 PMC6526822

[66] W. Feng , S. Huang , H. Wu , and M. Zhang , “Molecular Basis of Bcl‐xL's Target Recognition Versatility Revealed by the Structure of Bcl‐xL in Complex With the BH3 Domain of Beclin‐1,” Journal of Molecular Biology 372, no. 1 (September 2007): 223–235: Epub 2007 Jun 30. PMID: 17659302, 10.1016/j.jmb.2007.06.069. 17659302

[67] V. Pant , A. Quintás‐Cardama , and G. Lozano , “The p53 Pathway in Hematopoiesis: Lessons From Mouse Models, Implications for Humans,” Blood 120, no. 26 (December 2012): 5118–5127: Epub 2012 Sep 27. PMID: 23018641; PMCID: PMC3537308, 10.1182/blood-2012-05-356014. 23018641 PMC3537308

[68] J. Zhang , G. Kong , A. Rajagopalan , et al., “p53‐/‐ Synergizes With Enhanced NrasG12D Signaling to Transform Megakaryocyte‐Erythroid Progenitors in Acute Myeloid Leukemia,” Blood 129, no. 3 (January 2017): 358–370: Epub 2016 Nov 4. PMID: 27815262; PMCID: PMC5248933, 10.1182/blood-2016-06-719237. 27815262 PMC5248933

[69] R. Candau , D. M. Scolnick , P. Darpino , C. Y. Ying , T. D. Halazonetis , and S. L. Berger , “Two Tandem and Independent Sub‐Activation Domains in the Amino Terminus of p53 Require the Adaptor Complex for Activity,” Oncogene 15, no. 7 (August 1997): 807–816: PMID: 9266967, 10.1038/sj.onc.1201244. 9266967

[70] R. Ohki , T. Kawase , T. Ohta , H. Ichikawa , and Y. Taya , “Dissecting Functional Roles of p53 N‐Terminal Transactivation Domains by Microarray Expression Analysis,” Cancer Science 98, no. 2 (February 2007): 189–200: Erratum in: Cancer Sci. 2007 Mar;98(3):464. PMID: 17233836; PMCID: PMC11159457, 10.1111/j.1349-7006.2006.00375.x. 17233836 PMC11159457

[71] Y. Yin , C. W. Stephen , M. G. Luciani , and R. Fåhraeus , “p53 Stability and Activity is Regulated by Mdm2‐Mediated Induction of Alternative p53 Translation Products,” Nature Cell Biology 4, no. 6 (June 2002): 462–467: Erratum in: Nat Cell Biol 2002 Nov;4(11):912. PMID: 12032546, 10.1038/ncb801. 12032546

[72] G. Hofstetter , A. Berger , R. Berger , et al., “The N‐Terminally Truncated p53 Isoform Δ40p53 Influences Prognosis in Mucinous Ovarian Cancer,” International Journal of Gynecological Cancer 22, no. 3 (March 2012): 372–379: PMID: 22246403, 10.1097/IGC.0b013e31823ca031. 22246403

[73] W. A. Freed‐Pastor and C. Prives , “Mutant p53: One Name, Many Proteins,” Genes & Development 26, no. 12 (June 2012): 1268–1286: PMID: 22713868; PMCID: PMC3387655, 10.1101/gad.190678.112. 22713868 PMC3387655

[74] L. J. Valente , D. H. Gray , E. M. Michalak , et al., “p53 Efficiently Suppresses Tumor Development in the Complete Absence of Its Cell‐Cycle Inhibitory and Proapoptotic Effectors p21, Puma, and Noxa,” Cell Reports 3, no. 5 (May 2013): 1339–1345: Epub 2013 May 9. PMID: 23665218, 10.1016/j.celrep.2013.04.012. 23665218

[75] C. Lu and W. S. El‐Deiry , “Targeting p53 for Enhanced Radio‐ and Chemo‐Sensitivity,” Apoptosis 14, no. 4 (April 2009): 597–606: PMID: 19259822, 10.1007/s10495-009-0330-1. 19259822

[76] K. H. Vousden and X. Lu , “Live or Let Die: The Cell's Response to p53,” Nature Reviews Cancer 2, no. 8 (August 2002): 594–604: PMID: 12154352, 10.1038/nrc864. 12154352

[77] J. Milner and E. A. Medcalf , “Cotranslation of Activated Mutant P53 With Wild Type Drives the Wild‐Type p53 Protein Into the Mutant Conformation,” Cell 65, no. 5 (May 1991): 765–774: PMID: 2040013, 10.1016/0092-8674(91)90384-b. 2040013

[78] P. A. Muller and K. H. Vousden , “p53 Mutations in Cancer,” Nature Cell Biology 15, no. 1 (January 2013): 2–8: PMID: 23263379, 10.1038/ncb2641. 23263379

[79] K. Sabapathy , “The Contrived Mutant p53 Oncogene ‐ Beyond Loss of Functions,” Frontiers in Oncology 5 (December 2015): 276: Erratum in: Front Oncol. 2021 Mar 31;11:665504. doi: 10.3389/fonc.2021.665504. PMID: 26697411; PMCID: PMC4674554, 33869070 10.3389/fonc.2021.665504 PMC8047636

[80] M. E. Lomax , D. M. Barnes , T. R. Hupp , S. M. Picksley , and R. S. Camplejohn , “Characterization of p53 Oligomerization Domain Mutations Isolated From Li‐Fraumeni and Li‐Fraumeni Like Family Members,” Oncogene 17, no. 5 (August 1998): 643–649: PMID: 9704930, 10.1038/sj.onc.1201974. 9704930

[81] H. Seifert , B. Mohr , C. Thiede , et al., and Study Alliance Leukemia (SAL) , “The Prognostic Impact of 17p (p53) Deletion in 2272 Adults With Acute Myeloid Leukemia,” Leukemia 23, no. 4 (April 2009): 656–663: Epub 2009 Jan 8. PMID: 19151774, 10.1038/leu.2008.375. 19151774

[82] N. Cancer Genome Atlas Research , T. J. Ley , C. Miller , et al., “Genomic and Epigenomic Landscapes of Adult De Novo Acute Myeloid Leukemia,” New England Journal of Medicine 368, no. 22 (May 2013): 2059–2074: Epub 2013 May 1. Erratum in: N Engl J Med. 2013 Jul 4;369(1):98. PMID: 23634996; PMCID: PMC3767041, 10.1056/NEJMoa1301689. 23634996 PMC3767041

[83] K. T. Prochazka , G. Pregartner , F. G. Rücker , et al., “Clinical Implications of Subclonal TP53 Mutations in Acute Myeloid Leukemia,” Haematologica 104, no. 3 (March 2019): 516–523: Epub 2018 Oct 11. PMID: 30309854; PMCID: PMC6395341, 10.3324/haematol.2018.205013. 30309854 PMC6395341

[84] D. A. Sallman , R. Komrokji , C. Vaupel , et al., “Impact of TP53 Mutation Variant Allele Frequency on Phenotype and Outcomes in Myelodysplastic Syndromes,” Leukemia 30, no. 3 (March 2016): 666–673: Epub 2015 Oct 30. PMID: 26514544; PMCID: PMC7864381, 10.1038/leu.2015.304. 26514544 PMC7864381

[85] B. Ghimire , M. Zimmer , and V. Donthireddy , “TP53‐Mutated Acute Myeloid Leukemia: Review of Treatment and Challenges,” European Journal of Haematology 114, no. 6 (March 2025): 924–937, 10.1111/ejh.14404. 40035191

[86] N. G. Daver , S. Iqbal , C. Renard , et al., “Treatment Outcomes for Newly Diagnosed, Treatment‐Naïve TP53‐Mutated Acute Myeloid Leukemia: A Systematic Review and Meta‐Analysis,” Journal of Hematology & Oncology 16, no. 1 (March 2023): 19: PMID: 36879351; PMCID: PMC9990239, 10.1186/s13045-023-01417-5. 36879351 PMC9990239

[87] J. E. Lancet , G. L. Uy , J. E. Cortes , et al., “CPX‐351 (Cytarabine and Daunorubicin) Liposome for Injection Versus Conventional Cytarabine Plus Daunorubicin in Older Patients With Newly Diagnosed Secondary Acute Myeloid Leukemia,” Journal of Clinical Oncology 36, no. 26 (September 2018): 2684–2692: Epub 2018 Jul 19. PMID: 30024784; PMCID: PMC6127025, 10.1200/JCO.2017.77.6112. 30024784 PMC6127025

[88] A. D. Goldberg , C. Talati , P. Desai , et al., “TP53 Mutations Predict Poorer Responses to CPX‐351 in Acute Myeloid Leukemia,” supplement, Blood 132, no. S1 (2018): 1433, 10.1182/blood-2018-99-117772.

[89] J. Othman , C. Wilhelm‐Benartzi , R. Dillon , et al., “A Randomized Comparison of CPX‐351 and FLAG‐Ida in Adverse Karyotype AML and High‐Risk MDS: The UK NCRI AML19 Trial,” Blood Advances 7, no. 16 (August 2023): 4539–4549: PMID: 37171402; PMCID: PMC10425682, 10.1182/bloodadvances.2023010276. 37171402 PMC10425682

[90] M. Nieto , E. Samper , M. F. Fraga , G. González de Buitrago , M. Esteller , and M. Serrano , “The Absence of p53 is Critical for the Induction of Apoptosis by 5‐Aza‐2'‐Deoxycytidine,” Oncogene 23, no. 3 (January 2004): 735–743: PMID: 14737108, 10.1038/sj.onc.1207175. 14737108

[91] J. S. Welch , A. A. Petti , C. A. Miller , et al., “TP53 and Decitabine in Acute Myeloid Leukemia and Myelodysplastic Syndromes,” New England Journal of Medicine 375, no. 21 (November 2016): 2023–2036: PMID: 27959731; PMCID: PMC5217532, 10.1056/NEJMoa1605949. 27959731 PMC5217532

[92] N. J. Short , H. M. Kantarjian , S. Loghavi , et al., “Treatment With a 5‐Day versus a 10‐Day Schedule of Decitabine in Older Patients With Newly Diagnosed Acute Myeloid Leukaemia: A Randomised Phase 2 Trial,” Lancet Haematology 6, no. 1 (January 2019): e29–e37: Epub 2018 Dec 10. PMID: 30545576; PMCID: PMC6563344, 10.1016/S2352-3026(18)30182-0. 30545576 PMC6563344

[93] C. D. DiNardo , B. A. Jonas , V. Pullarkat , et al., “Azacitidine and Venetoclax in Previously Untreated Acute Myeloid Leukemia,” New England Journal of Medicine 383, no. 7 (August 2020): 617–629: PMID: 32786187, 10.1056/NEJMoa2012971. 32786187

[94] K. Kim , A. Maiti , S. Loghavi , et al., “Outcomes of TP53‐Mutant Acute Myeloid Leukemia With Decitabine and Venetoclax,” Cancer 127, no. 20 (October 2021): 3772–3781: Epub 2021 Jul 13. PMID: 34255353; PMCID: PMC10462434, 10.1002/cncr.33689. 34255353 PMC10462434

[95] T. Badar , A. Nanaa , E. Atallah , et al., “Comparing Venetoclax in Combination With Hypomethylating Agents to Hypomethylating Agent‐Based Therapies for Treatment Naive TP53‐Mutated Acute Myeloid Leukemia: Results From the Consortium on Myeloid Malignancies and Neoplastic Diseases (COMMAND),” Blood Cancer Journal 14, no. 1 (February 2024): 32: PMID: 38378617; PMCID: PMC10879201, 10.1038/s41408-024-01000-2. 38378617 PMC10879201

[96] C. D. DiNardo , I. S. Tiong , A. Quaglieri , et al., “Molecular Patterns of Response and Treatment Failure After Frontline Venetoclax Combinations in Older Patients With AML,” Blood 135, no. 11 (March 2020): 791–803: PMID: 31932844; PMCID: PMC7068032, 10.1182/blood.2019003988. 31932844 PMC7068032

[97] N. Gangat , I. Johnson , K. McCullough , et al., “Molecular Predictors of Response to Venetoclax Plus Hypomethylating Agent in Treatment‐Naïve Acute Myeloid Leukemia,” Haematologica 107, no. 10 (October 2022): 2501–2505: PMID: 35770533; PMCID: PMC9521222, 10.3324/haematol.2022.281214. 35770533 PMC9521222

[98] D. A. Pollyea , K. W. Pratz , A. H. Wei , et al., “Outcomes in Patients With Poor‐Risk Cytogenetics With or Without TP53 Mutations Treated With Venetoclax and Azacitidine,” Clinical Cancer Research 28, no. 24 (December 2022): 5272–5279: PMID: 36007102; PMCID: PMC9751752, 10.1158/1078-0432.CCR-22-1183. 36007102 PMC9751752

[99] D. A. Sallman , A. F. McLemore , A. L. Aldrich , et al., “TP53 Mutations in Myelodysplastic Syndromes and Secondary AML Confer an Immunosuppressive Phenotype,” Blood 136, no. 24 (December 2020): 2812–2823: PMID: 32730593; PMCID: PMC7731792, 10.1182/blood.2020006158. 32730593 PMC7731792

[100] N. Daver , G. Garcia‐Manero , S. Basu , et al., “Efficacy, Safety, and Biomarkers of Response to Azacitidine and Nivolumab in Relapsed/Refractory Acute Myeloid Leukemia: A Nonrandomized, Open‐Label, Phase II Study,” Cancer Discovery 9, no. 3 (March 2019): 370–383: Epub 2018 Nov 8. PMID: 30409776; PMCID: PMC6397669, 10.1158/2159-8290.CD-18-0774. 30409776 PMC6397669

[101] J. F. Zeidner , B. G. Vincent , A. Ivanova , et al., “Phase II Trial of Pembrolizumab After High‐Dose Cytarabine in Relapsed/Refractory Acute Myeloid Leukemia,” Blood Cancer Discovery 2, no. 6 (September 2021): 616–629: PMID: 34778801; PMCID: PMC8580622, 10.1158/2643-3230.BCD-21-0070. 34778801 PMC8580622

[102] F. Ravandi , R. Assi , N. Daver , et al., “Idarubicin, Cytarabine, and Nivolumab in Patients With Newly Diagnosed Acute Myeloid Leukaemia or High‐Risk Myelodysplastic Syndrome: A Single‐Arm, Phase 2 Study,” Lancet Haematology 6, no. 9 (September 2019): e480–e488: Epub 2019 Aug 7. PMID: 31400961; PMCID: PMC6778960, 10.1016/S2352-3026(19)30114-0. 31400961 PMC6778960

[103] A. M. Zeidan , I. Boss , C. L. Beach , et al., “A Randomized Phase 2 Trial of Azacitidine With or Without Durvalumab as First‐Line Therapy for Older Patients With AML,” Blood Advances 6, no. 7 (April 2022): 2219–2229: PMID: 34933333; PMCID: PMC9006260, 10.1182/bloodadvances.2021006138. 34933333 PMC9006260

[104] A. M. Zeidan , I. Boss , C. L. Beach , et al., “A Randomized Phase 2 Trial of Azacitidine With or Without Durvalumab as First‐Line Therapy for Higher‐Risk Myelodysplastic Syndromes,” Blood Advances 6, no. 7 (April 2022): 2207–2218: PMID: 34972214; PMCID: PMC9006291, 10.1182/bloodadvances.2021005487. 34972214 PMC9006291

[105] N. Acharya , C. Sabatos‐Peyton , and A. C. Anderson , “Tim‐3 Finds Its Place in the Cancer Immunotherapy Landscape,” Journal for ImmunoTherapy of Cancer 8, no. 1 (June 2020): e000911: PMID: 32601081; PMCID: PMC7326247, 10.1136/jitc-2020-000911. 32601081 PMC7326247

[106] A. M. Brunner , J. Esteve , K. Porkka , et al., “Phase Ib Study of Sabatolimab (MBG453), a Novel Immunotherapy Targeting TIM‐3 Antibody, in Combination With Decitabine or Azacitidine in High‐ or Very High‐Risk Myelodysplastic Syndromes,” American Journal of Hematology 99, no. 2 (February 2024): E32–E36: Epub 2023 Nov 22. PMID: 37994196, 10.1002/ajh.27161. 37994196

[107] M. Zeidan , J. Westermann , T. Kovacsovics , et al., “AML‐484 First Results of a Phase II Study (STIMULUS‐AML1) Investigating Sabatolimab + Azacitidine + Venetoclax in Patients With Newly Diagnosed Acute Myeloid Leukemia (ND AML),” supplement, Clinical Lymphoma Myeloma and Leukemia 22, no. S2 (October 2022): S255, 10.1016/S2152-2650(22)01303-9.

[108] B. I. Sikic , N. Lakhani , A. Patnaik , et al., “First‐in‐Human, First‐in‐Class Phase I Trial of the Anti‐CD47 Antibody Hu5F9‐G4 in Patients With Advanced Cancers,” Journal of Clinical Oncology 37, no. 12 (April 2019): 946–953: Epub 2019 Feb 27. PMID: 30811285; PMCID: PMC7186585, 10.1200/JCO.18.02018. 30811285 PMC7186585

[109] N. G. Daver , P. Vyas , S. Kambhampati , et al., “Tolerability and Efficacy of the Anticluster of Differentiation 47 Antibody Magrolimab Combined With Azacitidine in Patients With Previously Untreated AML: Phase Ib Results,” Journal of Clinical Oncology 41, no. 31 (November 2023): 4893–4904: Epub 2023 Sep 13. PMID: 37703506; PMCID: PMC10617926, 10.1200/JCO.22.02604. 37703506 PMC10617926

[110] J. F. Zeidner , D. A. Sallman , C. Récher , et al., “Magrolimab Plus Azacitidine vs Physician's Choice for Untreated TP53‐Mutated Acute Myeloid Leukemia: The ENHANCE‐2 Study,” Blood (February 2025): 2024027408: Epub ahead of print. PMID: 40009500, 10.1182/blood.2024027408.40009500

[111] G. Garcia‐Manero , A. Przespolewski , Y. Abaza , et al., “Evorpacept (ALX148), a CD47‐Blocking Myeloid Checkpoint Inhibitor, in Combination With Azacitidine and Venetoclax in Patients With Acute Myeloid Leukemia (ASPEN‐05): Results From Phase 1a Dose Escalation Part,” supplement, Blood 140, no. S1 (2022): 9046–9047, 10.1182/blood-2022-157606.

[112] A. A. Lane , J. S. Garcia , E. G. Raulston , et al., “Phase 1b Trial of Tagraxofusp in Combination With Azacitidine With or Without Venetoclax in Acute Myeloid Leukemia,” Blood Advances 8, no. 3 (February 2024): 591–602: PMID: 38052038; PMCID: PMC10837492, 10.1182/bloodadvances.2023011721. 38052038 PMC10837492

[113] J. Vadakekolathu , C. Lai , S. Reeder , et al., “TP53 Abnormalities Correlate With Immune Infiltration and Associate With Response to Flotetuzumab Immunotherapy in AML,” Blood Advances 4, no. 20 (October 2020): 5011–5024: PMID: 33057635; PMCID: PMC7594389, 10.1182/bloodadvances.2020002512. 33057635 PMC7594389

[114] D. Ali , K. Jönsson‐Videsäter , S. Deneberg , et al., “APR‐246 Exhibits Anti‐Leukemic Activity and Synergism With Conventional Chemotherapeutic Drugs in Acute Myeloid Leukemia Cells,” European Journal of Haematology 86, no. 3 (March 2011): 206–215: Epub 2011 Jan 11. PMID: 21114538, 10.1111/j.1600-0609.2010.01557.x. 21114538

[115] D. Ali , D. K. Mohammad , H. Mujahed , et al., “Anti‐leukaemic Effects Induced by APR‐246 Are Dependent on Induction of Oxidative Stress and the NFE2L2/HMOX1 Axis That Can be Targeted by PI3K and mTOR Inhibitors in Acute Myeloid Leukaemia Cells,” British Journal of Haematology 174, no. 1 (July 2016): 117–126: Epub 2016 Mar 15. PMID: 26991755, 10.1111/bjh.14036. 26991755

[116] R. Birsen , C. Larrue , J. Decroocq , et al., “APR‐246 Induces Early Cell Death by Ferroptosis in Acute Myeloid Leukemia,” Haematologica 107, no. 2 (February 2022): 403–416: PMID: 33406814; PMCID: PMC8804578, 10.3324/haematol.2020.259531. 33406814 PMC8804578

[117] T. Cluzeau , M. Sebert , R. Rahmé , et al., “Eprenetapopt Plus Azacitidine in *TP53*‐Mutated Myelodysplastic Syndromes and Acute Myeloid Leukemia: A Phase II Study by the Groupe Francophone des Myélodysplasies (GFM),” Journal of Clinical Oncology 39, no. 14 (May 2021): 1575–1583: Epub 2021 Feb 18. PMID: 33600210; PMCID: PMC8099409, 10.1200/JCO.20.02342. 33600210 PMC8099409

[118] Aprea Therapeutics . Aprea Therapeutics Announces Results of Primary Endpoint From Phase 3 Trial of Eprenetapopt in TP53 Mutant Myelodysplastic Syndromes (MDS) [Press Release]. Published December 28, 2020, https://ir.aprea.com/news‐releases/news‐release‐details/aprea‐therapeutics‐announces‐results‐primary‐endpoint‐phase‐3/.

[119] D. D. Fang , Q. Tang , Y. Kong , et al., “MDM2 Inhibitor APG‐115 Exerts Potent Antitumor Activity and Synergizes With Standard‐of‐Care Agents in Preclinical Acute Myeloid Leukemia Models,” Cell Death Discovery 7, no. 1 (May 2021): 90: PMID: 33941774; PMCID: PMC8093284, 10.1038/s41420-021-00465-5. 33941774 PMC8093284

[120] C. D. DiNardo , R. Olin , E. S. Wang , et al., “Phase 1 Dose Escalation Study of the MDM2 Inhibitor Milademetan as Monotherapy and in Combination With Azacitidine in Patients With Myeloid Malignancies,” Cancer Medicine 13, no. 14 (July 2024): e70028: PMID: 39030997; PMCID: PMC11258486, 10.1002/cam4.70028. 39030997 PMC11258486

[121] N. G. Daver , M. Dail , J. S. Garcia , et al., “Venetoclax and Idasanutlin in Relapsed/Refractory AML: A Nonrandomized, Open‐Label Phase 1b Trial,” Blood 141, no. 11 (March 2023): 1265–1276: PMID: 36265087; PMCID: PMC10651777, 10.1182/blood.2022016362. 36265087 PMC10651777

[122] M. Y. Konopleva , C. Röllig , J. Cavenagh , et al., “Idasanutlin Plus Cytarabine in Relapsed or Refractory Acute Myeloid Leukemia: Results of the MIRROS Trial,” Blood Advances 6, no. 14 (July 2022): 4147–4156: PMID: 35413116; PMCID: PMC9327534, 10.1182/bloodadvances.2021006303. 35413116 PMC9327534

[123] T. Badar , E. Atallah , R. Shallis , et al., “Survival of TP53‐Mutated Acute Myeloid Leukemia Patients Receiving Allogeneic Stem Cell Transplantation After First Induction or Salvage Therapy: Results From the Consortium on Myeloid Malignancies and Neoplastic Diseases (COMMAND),” Leukemia 37, no. 4 (April 2023): 799–806: Epub 2023 Feb 18. PMID: 36807649, 10.1038/s41375-023-01847-7. 36807649

[124] S. O. Ciurea , A. Chilkulwar , R. M. Saliba , et al., “Prognostic Factors Influencing Survival After Allogeneic Transplantation for AML/MDS Patients With *TP53* Mutations,” Blood 131, no. 26 (June 2018): 2989–2992: Epub 2018 May 16. PMID: 29769261; PMCID: PMC7218750, 10.1182/blood-2018-02-832360. 29769261 PMC7218750

[125] J. Loke , M. Labopin , C. Craddock , et al., “Additional Cytogenetic Features Determine Outcome in Patients Allografted for *TP53* Mutant Acute Myeloid Leukemia,” Cancer 128, no. 15 (August 2022): 2922–2931: Epub 2022 May 25. PMID: 35612815; PMCID: PMC9545190, 10.1002/cncr.34268. 35612815 PMC9545190

[126] R. C. Lindsley , W. Saber , B. G. Mar , et al., “Prognostic Mutations in Myelodysplastic Syndrome After Stem‐Cell Transplantation,” New England Journal of Medicine 376, no. 6 (February 2017): 536–547: PMID: 28177873; PMCID: PMC5438571, 10.1056/NEJMoa1611604. 28177873 PMC5438571

[127] B. Oran , M. de Lima , G. Garcia‐Manero , et al., “A Phase 3 Randomized Study of 5‐Azacitidine Maintenance vs Observation After Transplant in High‐Risk AML and MDS Patients,” Blood Advances 4, no. 21 (November 2020): 5580–5588: Erratum in: Blood Adv. 2021 Mar 23;5(6):1755‐1756. doi: 10.1182/bloodadvances.2021004374. PMID: 33170934; PMCID: PMC7656915, 33170934 10.1182/bloodadvances.2020002544 PMC7656915

[128] A. Mishra , R. Tamari , A. E. DeZern , et al., “Eprenetapopt Plus Azacitidine After Allogeneic Hematopoietic Stem‐Cell Transplantation for *TP53*‐Mutant Acute Myeloid Leukemia and Myelodysplastic Syndromes,” Journal of Clinical Oncology 40, no. 34 (December 2022): 3985–3993: Epub 2022 Jul 11. PMID: 35816664, 10.1200/JCO.22.00181. 35816664

[129] M. Shahzad , E. Tariq , S. G. Chaudhary , et al., “Outcomes With Allogeneic Hematopoietic Stem Cell Transplantation in *TP53*‐Mutated Acute Myeloid Leukemia: A Systematic Review and Meta‐Analysis,” Leukemia and Lymphoma 63, no. 14 (December 2022): 3409–3417: Epub 2022 Sep 15. PMID: 36107118, 10.1080/10428194.2022.2123228. 36107118

[130] S. Chen , J. L. Wu , Y. Liang , et al., “Arsenic Trioxide Rescues Structural P53 Mutations Through a Cryptic Allosteric Site,” Cancer Cell 39, no. 2 (February 2021): 225–239.e8: Epub 2020 Dec 24. PMID: 33357454, 10.1016/j.ccell.2020.11.013. 33357454

[131] G. J. Roboz , E. K. Ritchie , T. Curcio , et al., “Arsenic Trioxide and Low‐Dose Cytarabine in Older Patients With Untreated Acute Myeloid Leukemia, Excluding Acute Promyelocytic Leukemia,” Cancer 113, no. 9 (November 2008): 2504–2511: PMID: 18825661, 10.1002/cncr.23855. 18825661

[132] C. Floquet , J. Deforges , J. P. Rousset , and L. Bidou , “Rescue of Non‐Sense Mutated p53 Tumor Suppressor Gene by Aminoglycosides,” Nucleic Acids Research 39, no. 8 (April 2011): 3350–3362: Epub 2010 Dec 10. PMID: 21149266; PMCID: PMC3082906, 10.1093/nar/gkq1277. 21149266 PMC3082906

[133] L. Bidou , O. Bugaud , V. Belakhov , T. Baasov , and O. Namy , “Characterization of New‐Generation Aminoglycoside Promoting Premature Termination Codon Readthrough in Cancer Cells,” RNA Biology 14, no. 3 (March 2017): 378–388: Epub 2017 Feb 1. PMID: 28145797; PMCID: PMC5367250, 10.1080/15476286.2017.1285480. 28145797 PMC5367250

[134] J. P. Gudikote , T. Cascone , A. Poteete , et al., “Inhibition of Nonsense‐Mediated Decay Rescues p53β/γ Isoform Expression and Activates the P53 Pathway in MDM2‐Overexpressing and Select p53‐Mutant Cancers,” Journal of Biological Chemistry 297, no. 5 (November 2021): 101163: Epub 2021 Sep 3. PMID: 34481841; PMCID: PMC8569473, 10.1016/j.jbc.2021.101163. 34481841 PMC8569473

[135] E. A. Chapeau , A. Gembarska , E. Y. Durand , et al., “Resistance Mechanisms to TP53‐MDM2 Inhibition Identified by In Vivo piggyBac Transposon Mutagenesis Screen in an Arf‐/‐ Mouse Model,” Proceedings of the National Academy of Sciences 114, no. 12 (March 2017): 3151–3156: Epub 2017 Mar 6. PMID: 28265066; PMCID: PMC5373361, 10.1073/pnas.1620262114. PMC5373361 28265066

[136] D. Takahashi , J. Moriyama , T. Nakamura , et al., “AUTACs: Cargo‐Specific Degraders Using Selective Autophagy,” Molecular Cell 76, no. 5 (December 2019): 797–810.e10: Epub 2019 Oct 9. PMID: 31606272, 10.1016/j.molcel.2019.09.009. 31606272

[137] F. G. Rücker , K. M. Lang , M. Fütterer , et al., “Molecular Dissection of Valproic Acid Effects in Acute Myeloid Leukemia Identifies Predictive Networks,” Epigenetics 11, no. 7 (July 2016): 517–525: Epub 2016 Jun 16. PMID: 27309669; PMCID: PMC4939918, 10.1080/15592294.2016.1187350. 27309669 PMC4939918

[138] G. Li , J. Yao , Z. Lu , et al., “Simvastatin Preferentially Targets FLT3/ITD Acute Myeloid Leukemia by Inhibiting MEK/ERK and p38‐MAPK Signaling Pathways,” Drugs in R & D 23, no. 4 (December 2023): 439–451: Epub 2023 Oct 17. PMID: 37847357; PMCID: PMC10676344, 10.1007/s40268-023-00442-6. 37847357 PMC10676344

[139] C. Tognon , S. Mishra , A. Kaempf , et al., “Phase 1 Trial Testing the Novel Combination Therapy of Entrectinib and ASTX727 in TP53 Mutated Relapsed/Refractory Acute Myeloid Leukemia Patients,” supplement, Blood 144, no. S1 (2024): 1498, 10.1182/blood-2024-208505.

[140] A. Fujii , T. Masuda , M. Iwata , et al., “The Novel Driver Gene ASAP2 is a Potential Druggable Target in Pancreatic Cancer,” Cancer Science 112, no. 4 (April 2021): 1655–1668: Epub 2021 Mar 5. PMID: 33605496; PMCID: PMC8019229, 10.1111/cas.14858. 33605496 PMC8019229

[141] M. Kwok , N. Davies , A. Agathanggelou , et al., “ATR Inhibition Induces Synthetic Lethality and Overcomes Chemoresistance in *TP53*‐ or *ATM*‐Defective Chronic Lymphocytic Leukemia Cells,” Blood 127, no. 5 (February 2016): 582–595: Epub 2015 Nov 12. PMID: 26563132, 10.1182/blood-2015-05-644872. 26563132

[142] X. Cao , H. Dai , Q. Cui , et al., “CD7‐directed CAR T‐Cell Therapy: A Potential Immunotherapy Strategy for Relapsed/Refractory Acute Myeloid Leukemia,” Experimental Hematology & Oncology 11, no. 1 (September 2022): 67: PMID: 36175988; PMCID: PMC9523980, 10.1186/s40164-022-00318-6. 36175988 PMC9523980

[143] K. N. Kremer , K. L. Peterson , P. A. Schneider , et al., “CXCR4 Chemokine Receptor Signaling Induces Apoptosis in Acute Myeloid Leukemia Cells via Regulation of the Bcl‐2 Family Members Bcl‐XL, Noxa, and Bak,” Journal of Biological Chemistry 288, no. 32 (August 2013): 22899–22914: Epub 2013 Jun 24. PMID: 23798675; PMCID: PMC3743469, 10.1074/jbc.M113.449926. 23798675 PMC3743469

